# Retinal Microenvironment‐Protected Rhein‐GFFYE Nanofibers Attenuate Retinal Ischemia‐Reperfusion Injury via Inhibiting Oxidative Stress and Regulating Microglial/Macrophage M1/M2 Polarization

**DOI:** 10.1002/advs.202302909

**Published:** 2023-08-31

**Authors:** Zhuhong Zhang, Shengjun Peng, Tengyan Xu, Jia Liu, Laien Zhao, Hui Xu, Wen Zhang, Yuanying Zhu, Zhimou Yang

**Affiliations:** ^1^ School of Pharmacy Key Laboratory of Molecular Pharmacology and Drug Evaluation Ministry of Education Collaborative Innovation Center of Advanced Drug Delivery System and Biotech Drugs in Universities of Shandong Yantai University Yantai 264005 China; ^2^ Key Laboratory of Bioactive Materials Ministry of Education State Key Laboratory of Medicinal Chemical Biology College of Life Sciences Collaborative Innovation Center of Chemical Science and Engineering and National Institute of Functional Materials Nankai University Tianjin 300071 China

**Keywords:** oxidative stress, retinal ischemia‐reperfusion injury, retinal microenvironment, rhein, self‐assembling peptides

## Abstract

Retinal ischemia is involved in the occurrence and development of various eye diseases, including glaucoma, diabetic retinopathy, and central retinal artery occlusion. To the best of our knowledge, few studies have reported self‐assembling peptide natural products for the suppression of ocular inflammation and oxidative stress. Herein, a self‐assembling peptide GFFYE is designed and synthesized, which can transform the non‐hydrophilicity of rhein into an amphiphilic sustained‐release therapeutic agent, and rhein‐based therapeutic nanofibers (abbreviated as Rh‐GFFYE) are constructed for the treatment of retinal ischemia‐reperfusion (RIR) injury. Rh‐GFFYE significantly ameliorates oxidative stress and inflammation in an in vitro oxygen‐glucose deprivation (OGD) model of retinal ischemia and a rat model of RIR injury. Rh‐GFFYE also significantly enhances retinal electrophysiological recovery and exhibits good biocompatibility. Importantly, Rh‐GFFYE also promotes the transition of M1‐type macrophages to the M2 type, ultimately altering the pro‐inflammatory microenvironment. Further investigation of the treatment mechanism indicates that Rh‐GFFYE activates the PI3K/AKT/mTOR signaling pathway to reduce oxidative stress and inhibits the NF‐κB and STAT3 signaling pathways to affect inflammation and macrophage polarization. In conclusion, the rhein‐loaded nanoplatform alleviates RIR injury by modulating the retinal microenvironment. The findings are expected to promote the clinical application of hydrophobic natural products in RIR injury‐associated eye diseases.

## Graphical abstract

Current clinical treatments for retinal ischemia‐related diseases focus on a single pathological mechanism. Despite the anti‐inflammatory and antioxidant pharmacological activities of rhein, its non‐hydrophilicity limits the potential clinical application. This study constructs a peptide‐based rhein nanoplatform and proposes that it can alleviate RIR injury by modulating the retinal microenvironment.

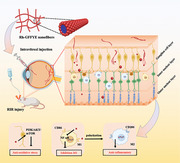

## 1 Introduction

Retinal ischemia‐reperfusion (RIR) injury is a common cause of various ocular disorders such as glaucoma, diabetic retinopathy, retinal vascular occlusions, and retinopathy of prematurity.^[^ ^1^ ^]^ Currently, few effective treatments can reverse the blockage caused by retinal vascular occlusions.^[^ ^1a^ ^]^ Glaucoma, one of hallmarks of which is pathologically elevated intraocular pressure (IOP), is prone to damage retinal ganglion cells (RGCs).^[^ ^2^ ^]^ The main treatment strategies to reduce IOP are laser therapy, surgery, and eye drops.^[^ ^3^ ^]^ The clinical treatment of diabetic retinopathy characterized by pathological angiogenesis is mainly based on the intravitreal injection of anti‐VEGF drugs.^[^ ^4^ ^]^ However, these treatments either have poor patient compliance or are prone to result in intraocular inflammation due to repeated injections. These limitations have prompted the investigation of alternatives with greater safety margins and better ability to treat RIR injury‐related diseases.^[^ ^5^ ^]^

RIR injury can lead to a variety of pathological biological events, including oxidative stress, inflammation, and RGCs death, resulting in changes in the retinal microenvironment.^[^ ^6^ ^]^ An early event in RIR injury is the overproduction of reactive oxygen species (ROS) that induce oxidative stress. This process can also activate the toll‐like receptor 4 (TLR4)/ nuclear factor‐κB (NF‐κB) signaling pathway, thereby triggering inflammation that ultimately leads to the loss of RGCs.^[^ ^7^ ^]^ External stimuli can activate the phosphatidylinositol 3‐kinase (PI3K)/protein kinase B (AKT) pathway to phosphorylate the downstream signaling molecule target of rapamycin (mTOR), thereby inhibiting oxidative stress and inflammation. In the inflammatory microenvironment of multiple organs, microglia/macrophages can be polarized into two functional phenotypes: M1 (pro‐inflammatory) and M2 (anti‐inflammatory).^[^ ^8^ ^]^ Recent studies have shown that promoting the transition of microglia/macrophages from the M1 phenotype to the M2 phenotype attenuates RIR injury.^[^ ^9^ ^]^ Moreover, the activation of the signal transducer and activator of transcription‐3 (STAT3) signaling pathway has been shown to facilitate M1 microglia/macrophage polarization and enhance the expressions of inflammatory cytokines.^[^ ^10^ ^]^ However, current clinical treatments for retinal ischemia‐related diseases focus on a single pathological mechanism. Therefore, protecting ​the retinal microenvironment by modulating multiple signaling pathways shows promise as a therapeutic approach for RIR injury.

Herb‐derived natural products are regarded as promising drug resources due to their anti‐inflammatory and antioxidant effects.^[^ ^11^ ^]^ Rhein (4,5‐dihydroxyanthraquinone‐2‐carboxylic acid), is an anthraquinone and one of the pharmacologically active components extracted from *Rheum*.^[^ ^12^ ^]^ Rhein has been shown to significantly reduce the ROS level, reverse the depletion of mitochondrial membrane potential, and protect neurons from oxidative stress‐associated apoptosis.^[^ ^13^ ^]^ Due to its good anti‐apoptotic and anti‐inflammatory effects, rhein has also been used to treat ischemia/reperfusion diseases of various organs, including the brain and heart.^[^ ^14^ ^]^ Moreover, rhein was reported to attenuate intestinal inflammatory responses by modulating macrophage polarization from the M1 phenotype to the M2 phenotype.^[^ ^15^ ^]^ To the best of our knowledge, studies on the effects of rhein on retinal ischemia are limited. Rhein is a natural product with characteristics that make it unsuitable for direct intravitreal administration for the treatment of retinal ischemia. Thus, loading rhein into an amphiphilic drug delivery system is conducive to its clinical application. Zhao et al. prepared a supramolecular hydrogel to transport rhein for the treatment of chronic wounds.^[^ ^16^ ^]^ In addition to rhein, the supramolecular hydrogel also contained hyaluronic acid, ferrocene, and β‐cyclodextrin. Zheng et al. reported that rhein can self‐assemble into a translucent hydrogel at a concentration of 14.1 mM and a pH between 8.0 and 9.4.^[^ ^17^ ^]^ However, the eye is a sensitive organ, and the pH of the vitreous humor is ≈ 7.8. Alkaline pH and excess chemical materials in the carrier may cause ocular toxicity. Proteins and peptides are the main components of organisms in nature and participate in numerous biological processes. Therefore, self‐assembling peptides have great potential in the treatment of ocular diseases due to their excellent biocompatibility and bioactivity, easy modification, and responsiveness to biological signals.^[^ ^18^ ^]^ Small molecules can be covalently linked to peptides to produce nanosystems that have high, fixed, and tunable drug loading; importantly, these nanosystems are hydrophilic and show sustained release ability.^[^ ^18b^ ^]^ In a previous study, we designed and synthesized novel rhein‐GFFYERGD (Rh‐GFFYERGD) nanofibers (pH 7.4) that can be chelated with cisplatin to form hydrogels exhibiting excellent nuclear aggregation and antitumor properties.^[^ ^19^ ^]^ Based on the pharmacological activity of rhein and the findings of our previous work, we hypothesized that self‐assembled rhein peptide nanofibers would exert good antioxidant and anti‐inflammatory effects in RIR injury.

**Scheme 1:**
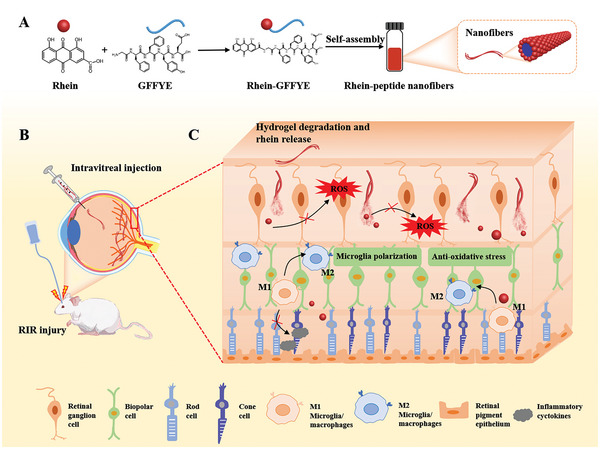
Schematic representation of Rh‐GFFYE as a therapeutic agent for RIR injury. A) Schematic diagram of the nanostructure of Rh‐GFFYE. B) The established RIR injury model and intravitreal injection of Rh‐GFFYE. C) Rh‐GFFYE reprograms macrophage phenotypes, relieves inflammation, protects retinal cells, and eliminates ROS.

In this study, we evaluated the neuroprotective effect of Rh‐GFFYE nanofibers in RIR injury along with their ability to regulate the retinal microenvironment after RIR injury. We also characterized the distribution and safety of the Rh‐GFFYE nanofibers in the retina and explored the underlying molecular mechanism of Rh‐GFFYE in retinal protection (**Scheme** **1**).

## 2 Results

### 2.1 Molecular Formula and Characterization of Rh‐GFFYE and Control Compounds

**Figure 1:**
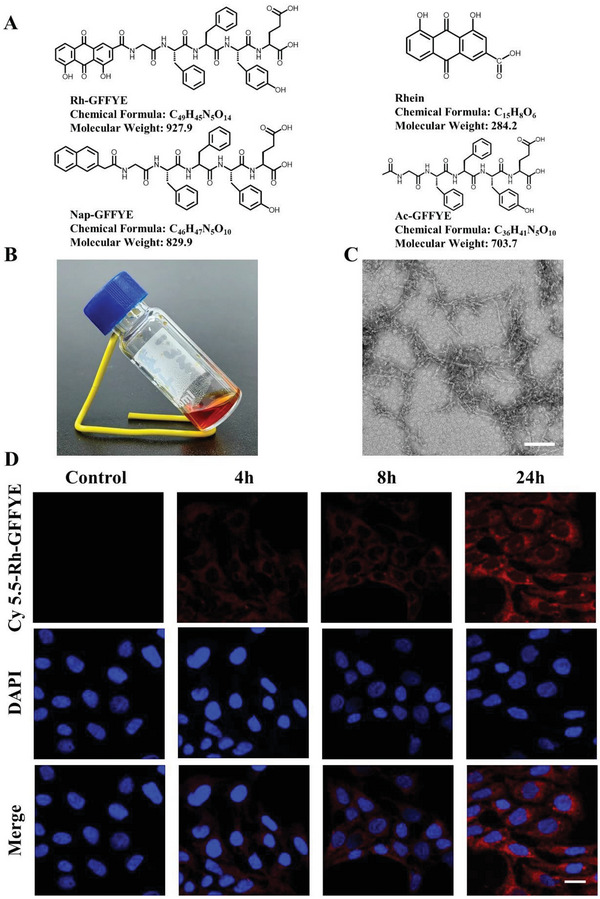
Characterization and in vitro uptake of Rh‐GFFYE. A) The structural formula, chemical formula, and molecular weight of Rh‐GFFYE, rhein, Nap‐GFFYE and Ac‐GFFYE. B) Optical image of the Rh‐GFFYE nanofibers. C) Rh‐GFFYE nanofibers observed by TEM. Scale bar = 100 nm. D) CLSM images of time‐dependent cellular uptake of Cy5.5‐Rh‐GFFYE (red fluorescence) in R28 cells after 4, 8, and 24 h of incubation. Scale bar = 25 µm.

In our previous study, we developed Rh‐GFFYERGD nanofibers that form anti‐cancer nanomedicines with cisplatin.^[^ ^19^ ^]^ In the present study, we further explored the role of rhein in RIR injury. Two control peptide compounds were synthesized: Nap‐GFFYE, in which rhein is replaced with 1‐naphthylacetic acid (Nap); and Ac‐GFFYE, which retains the peptide sequence. **Figure** **1**A shows the chemical information of all compounds. The molecular structures of compounds were confirmed by ^1^H NMR (Figure S1, Supporting Information) and mass spectrometry (MS, Figure S2, Supporting Information). At a concentration of 0.4 wt.%, Rh‐GFFYE forms a clear solution in phosphate‐buffered saline (PBS) with a pH of 7.4 (Figure 1B). As shown by the transmission electron microscopy (TEM) image in Figure 1C, Rh‐GFFYE formed short nanofibers with diameters of ≈10 nm. These results demonstrate the successful design and synthesis of Rh‐GFFYE, a nanoplatform loaded with the natural product rhein that has potential clinical significance for the treatment of RIR injury.

Retinal cell line R28, an adherent retinal precursor cell line suitable for experimental retinal transplantation and in vitro studies of retinal cell activity, has previously been used in studies of ocular diseases.^[^ ^7^, ^20^ ^]^ To explore whether Rh‐GFFYE can effectively deliver rhein into cells to exert therapeutic effects, the cellular internalization of the Rh‐GFFYE nanofibers in R28 cells was investigated using Cyanine5.5 (Cy5.5)‐labeled Rh‐GFFYE (Cy5.5‐Rh‐GFFYE). We confirmed that Rh‐GFFYE was internalized by R28 cells. As shown in Figure 1D, time‐dependent fluorescence signals were detected in R28 cells after incubation with Cy5.5‐Rh‐GFFYE. Obvious intracellular fluorescence appeared when R28 cells were treated with 20 µM Cy5.5‐Rh‐GFFYE for 4 h, indicating the extremely rapid cellular uptake of a large quantity of nanofibers.

### 2.2 Rh‐GFFYE Improves Cell Viability and Attenuates Oxidative Stress in R28 Cells Subjected to Oxygen‐Glucose Deprivation (OGD)

**Figure 2:**
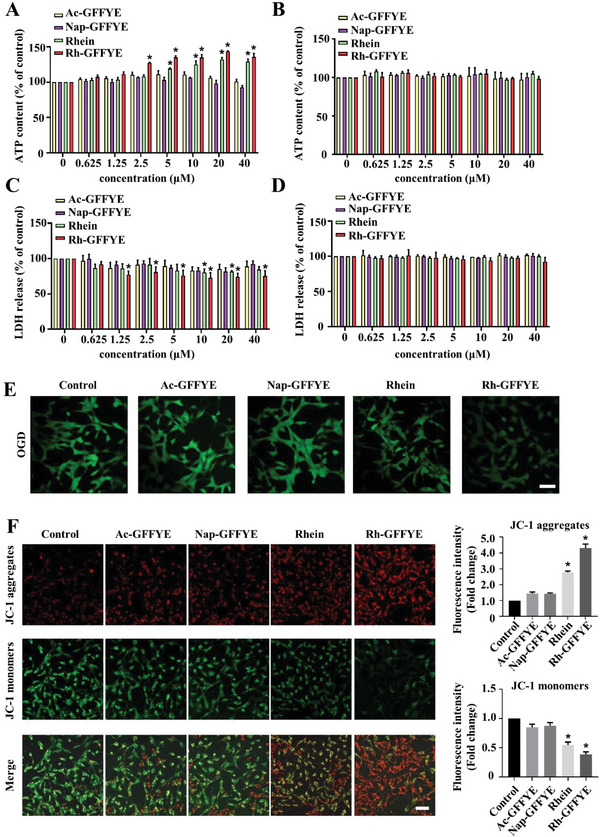
Rh‐GFFYE protects R28 cells subject to OGD. A) Cellular ATP content and C) LDH release showing the dose‐dependent effect of Rh‐GFFYE on OGD‐induced cytotoxicity in R28 cells. Rh‐GFFYE showed no toxicity based on cellular ATP B) and LDH release D). E) Levels of ROS in R28 cells were monitored by the H_2_DCF‐DA fluorescent probe. Scale bar = 50 µm. F) Mitochondrial membrane potential (ΔΨm) staining in R28 cells were monitored by JC‐1 fluorescent prob. Quantification of fluorescence intensity of JC‐1 aggregates (red fluorescence) and JC‐1 monomers (green fluorescence) by Image J software. Scale bar = 50 µm. The data were presented as the mean ± SD of *n* = 3; *p< 0.05 compared with the Control group.

In this study, the in vitro antioxidant efficacy of Rh‐GFFYE was examined using the OGD model, a widely reported model for ischemia‐reperfusion injury in multiple organs including the brain and retina.^[^ ^7^, ^21^ ^]^ We validated the hypothesis that Rh‐GFFYE can rescue R28 cells from OGD‐induced cell death. R28 cells pretreated for 24 h with varying concentrations of drugs (Rh‐GFFYE, rhein, Nap‐GFFYE, and Ac‐GFFYE) were subjected to OGD. The adenosine triphosphate (ATP) contents shown in **Figure** **2**A demonstrate that treatment with Rh‐GFFYE at doses of 10, 20, and 40 µM resulted in the significant recovery of cell viability. The best effect was obtained at the Rh‐GFFYE concentration of 20 µM, which restored the ATP content by ≈ 40% compared with the control. Treatment with rhein, Nap‐GFFYE, or Ac‐GFFYE at all concentrations showed no restorative effect on R28 cells subjected to OGD compared with the Rh‐GFFYE group. To further verify the protective effect of Rh‐GFFYE, we examined whether Rh‐GFFYE could attenuate the increase in lactate dehydrogenase (LDH) release caused by OGD. As shown in Figure 2C, LDH release was inhibited in the presence of Rh‐GFFYE. Among the experimental groups, Rh‐GFFYE had the best inhibition effect on LDH release. For safety evaluation, R28 cells were treated with Rh‐GFFYE and other drugs under normoxic conditions for 24 h, and the levels of ATP and LDH were then detected. The results show that Rh‐GFFYE had no toxic effect on R28 cells (Figure 2B,D). The above results demonstrate that OGD causes oxidative stress in R28 cells, and Rh‐GFFYE treatment can save the R28 cells from oxidative stress, as indicated by the recovery of ATP level and the decrease in LDH release.

As previously reported, intracellular ROS production is dramatically elevated under OGD conditions.^[^ ^22^ ^]^ Thus, we also evaluated the effects of Rh‐GFFYE and the control compounds on the intracellular ROS level. The cellular ROS levels in R28 cells were assessed by 2′,7′‐dichlorofluorescin diacetate (H_2_DCF‐DA) assay using confocal laser scanning microscope (CLSM) observation (Figure 2E). As expected, OGD stimulation alone clearly induced ROS overproduction in R28 cells, as evidenced by the high intensity of green fluorescence. Treatment with Rh‐GFFYE or rhein resulted in significant ROS inhibition compared with the control for R28 cells exposed to OGD. It is worth noting that compared with rhein, Rh‐GFFYE showed a stronger ROS inhibition effect. Considering our results, we can conclude that Rh‐GFFYE possesses good antioxidant capacity and has the potential to relieve RIR injury.

The decrease in the mitochondrial membrane potential (ΔΨm) is a landmark event in the early stage of apoptosis.^[^ ^23^ ^]^ 5,5',6,6'‐Tetrachloro‐1,1',3,3'‐tetraethyl‐imidacarbocyanine iodide (JC‐1) is commonly used to monitor ΔΨm. When ΔΨm is high, JC‐1 aggregates in the mitochondrial matrix, forming J‐aggregates and producing red fluorescence; in contrast, when ΔΨm is low, JC‐1 cannot aggregate in the mitochondrial matrix. In this case, JC‐1 is a monomer that produces green fluorescence. As shown in Figure 2F, JC‐1 fluorescence staining demonstrated that the ΔΨm value of R28 cells was obviously decreased under OGD stimulation and recovered after treatment with Rh‐GFFYE or rhein. In contrast, no recovery in ΔΨm was observed after treatment with Nap‐GFFYE or Ac‐GFFYE. In addition, Rh‐GFFYE had a more significant protective effect on the ΔΨm value of R28 cells compared with rhein, consistent with our earlier results.

Taken together, these results suggest that Rh‐GFFYE has a strong antioxidant capacity and can restore the viability of R28 cells under OGD, reduce ROS production, and stabilize the mitochondrial membrane potential.

### 2.3 Rh‐GFFYE Alleviates LPS‐induced Inflammation in Macrophages

**Figure 3:**
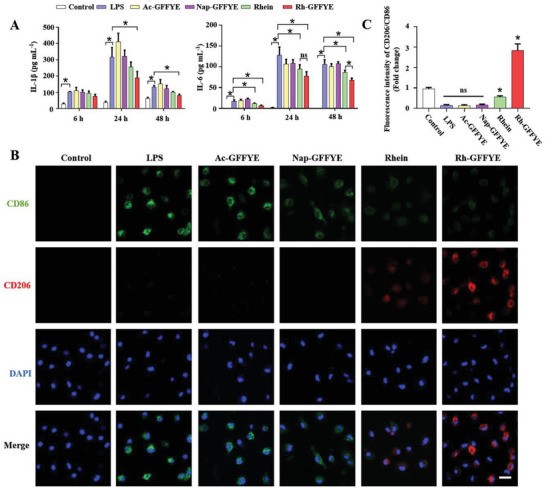
Rh‐GFFYE attenuates LPS‐induced inflammation in macrophages. A) Effect of Rh‐GFFYE reduced LPS‐induced IL‐1β and IL‐6 cytokine levels in BMDMs. Data were presented as mean ± SD from three independent experiments. *p < 0.05 compared with the LPS group. B) Representative CLSM images of M1 (FITC‐CD86, green) and M2 (PE‐CD206, red) BMDMs repolarization after Rh‐GFFYE treatment. Treatment with Rh‐GFFYE significantly reduced the population of M1 macrophages (CD86^+^ cells) and increased the population of M2 macrophages (CD206^+^ cells). Nuclei were stained with DAPI (blue). Scale bar = 20 µm. C) Fluorescence intensities of CD206/CD86 quantified by Image J software (*n* = 6). *p < 0.05 compared with the LPS group.

To investigate the ability of Rh‐GFFYE to attenuate inflammation, we measured the levels of inflammatory cytokines using enzyme‐linked immunosorbent assay (ELISA). We first used lipopolysaccharide (LPS)‐induced bone marrow‐derived macrophages (BMDMs) as an in vitro model to estimate the anti‐inflammatory effects of Rh‐GFFYE. Compared with the Control group, LPS resulted in a significant accumulation of interleukin‐1β (IL‐1β) and interleukin‐6 (IL‐6), and their levels peaked at 24 h after treatment (**Figure** **3**A). At 6 h after treatment, Rh‐GFFYE had significantly reduced the level of IL‐6, whereas the level of IL‐1β was not markedly altered. At 24 h after treatment, the IL‐1β and IL‐6 levels were both approximately two times lower in the Rh‐GFFYE group than in the LPS‐stimulated group. Rhein also reduced the release of these two inflammatory cytokines to some extent, but the effect was weaker than that of Rh‐GFFYE. In contrast, Nap‐GFFYE and Ac‐GFFYE did not show anti‐inflammatory effects. The trends in the levels of inflammatory cytokines at 48 h after treatment were similar to those observed at 24 h; for example, the levels of both IL‐1β and IL‐6 were approximately two times lower in the Rh‐GFFYE treatment group than in the LPS‐induced group.

Rhein has been reported to inhibit inflammatory response while reprogramming the macrophage phenotype.^[^ ^16^, ^24^ ^]^ To further explore the anti‐inflammatory activity of Rh‐GFFYE, we examined the effect of Rh‐GFFYE on the polarization of BMDMs. To confirm the change in macrophage phenotype, macrophage surface markers (CD86 for the M1 phenotype and CD206 for the M2 phenotype) were detected by CLSM. As shown in Figure 3B,C, LPS significantly enhanced the expression of M1 macrophages, as reflected by the higher intensity of green fluorescence (CD86^+^ cells) compared with the Control group. After treatment with Rh‐GFFYE, the number of CD86^+^ cells decreased, accompanied by enhanced red fluorescence intensity (CD206^+^ cells), confirming the transformation of macrophages to the M2 phenotype. These results indicate that Rh‐GFFYE inhibits inflammatory response in part by transforming macrophages from the M1 phenotype to the M2 phenotype.

### 2.4 Rh‐GFFYE Attenuates RIR injury

**Figure 4:**
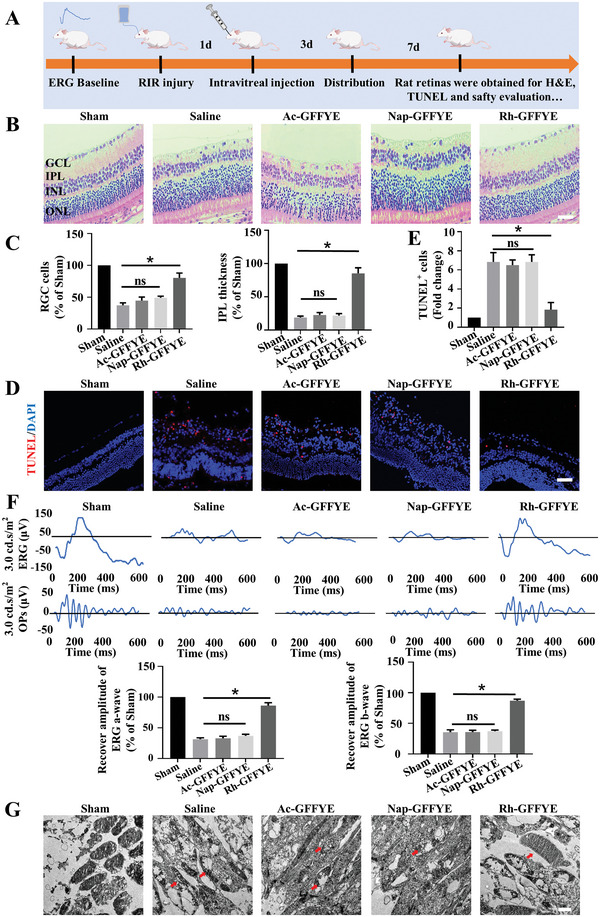
Intravitreal injection of Rh‐GFFYE protects RGCs and retinal function after RIR injury in vivo. A) Experimental schedule for the intravitreal injection of Rh‐GFFYE to treat RIR injury. B) H&E images of retinal paraffin sections in different groups. Scale bar = 50 µm; *n* = 6; GCL, ganglion cell layer; IPL, inner plexiform layer; INL, inner nuclear layer; ONL, outer nuclear layer. C) Retinas exposed to RIR injury showed significant RGC loss and decreased IPL thickness. D) Representative TUNEL staining images. Scale bar = 50 µm; *n* = 6. E) Intravitreal injection of Rh‐GFFYE significantly reduced TUNEL^+^ cells. F) Representative ERG traces at 3.00 cd∙s∙m^−2^ from rats in the Sham, Saline, Ac‐GFFYE, Nap‐GFFYE, and Rh‐GFFYE groups along with the amplitudes of the scotopic ERG a and b waves in the Sham, Saline, Ac‐GFFYE, Nap‐GFFYE, and Rh‐GFFYE groups at a light intensity of 3.00 cd∙s∙m^−2^. G) TEM images showing the morphologies of mitochondria in retinal tissues from the Sham, Saline, Ac‐GFFYE, Nap‐GFFYE, and Rh‐GFFYE groups. Scale bar = 1 µm. The data were presented as mean ± SD (*n* = 6). *p < 0.05 compared with the Saline group.

The therapeutic effect of Rh‐GFFYE was further investigated in vivo by establishing a rat model of RIR injury.^[^ ^7b^ ^]^ The experimental schedule is shown in **Figure** **4**A. After the model was established, the rats were intravitreally injected to mimic the clinical drug delivery strategy for ocular diseases.^[^ ^25^ ^]^ Oxidative stress and inflammation during RIR injury eventually lead to apoptosis in RGCs and a reduction in the thickness of the inner plexiform layer (IPL).^[^ ^26^ ^]^ As shown in Figure 4B, after RIR injury, the thickness of the IPL in the RIR/Saline group was significantly reduced to 20% of that in the Sham group. Treatment with Ac‐GFFYE or Nap‐GFFYE did not ameliorate this decrease in IPL thickness caused by RIR injury (Figure 4C). In contrast, Rh‐GFFYE treatment reversed the RIR injury‐induced decrease in IPL thickness and reduced the apoptosis of RGCs (Figure 4C). In our study, TUNEL staining indicated that Rh‐GFFYE administration significantly decreased RIR injury‐induced apoptosis in retinal cells (Figure 4D,E).

Electroretinography (ERG), which reflects retinal function, was performed on the anesthetized animals after overnight dark adaptation.^[^ ^27^ ^]^ The ERG results were normalized to the baseline prior to ischemia and to control eyes. As shown in Figure 4F, the a and b wave amplitudes showed significant recovery at 7 d after the intravitreal injection of Rh‐GFFYE. The a and b waves in the Rh‐GFFYE group were not significantly different from those in the Sham group. In contrast, the a and b wave amplitudes were not restored in the other treatment groups. The oscillatory potentials (OPs), which are thought to arise within the IPL, were also monitored during ERG in the present study.^[^ ^28^ ^]^ After RIR injury, the amplitudes of the OPs in the RIR/Saline group were significantly reduced due to the thinning of the IPL layer. Meanwhile, the amplitudes of the OPs were recovered in the Rh‐GFFYE‐treated group, as indicated by the significantly higher sum of OPs amplitudes compared with the RIR/Saline group (Figure 4F). We also evaluated the mitochondrial morphology after RIR injury by TEM. Consistent with our previous findings, the mitochondrial membrane in the RIR/Saline group showed significant morphological changes, and the administration of Rh‐GFFYE partially reversed this effect (Figure 4G).^[^ ^29^ ^]^

### 2.5 Rh‐GFFYE Administration Following Retinal Ischemia In Vivo Attenuates the Loss of RGCs

**Figure 5:**
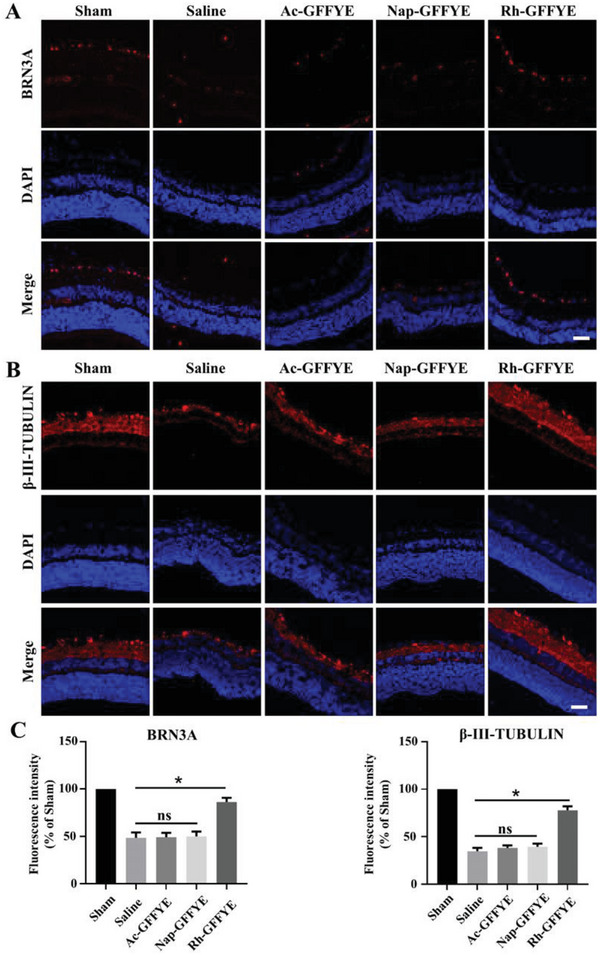
Intravitreal injection of Rh‐GFFYE reverses the damage to RGCs caused by RIR injury. Representative CLSM images of RGCs markers, BRN3A A) and β‐III‐TUBULIN B) in retinal cryosections collected at 7 days after intravitreal injection. Scale bar = 50 µm. C). BRN3A and β‐III‐TUBULIN fluorescence intensity were quantified using Image J software. The data were presented as the mean ± SD of *n* = 6; *p < 0.05 compared with the Saline group.

Brain‐specific homebox/POU domain protein 3A (BRN3A) and β‐III‐TUBULIN are two protein markers of RGC cells that can be labeled using immunofluorescence staining.^[^ ^23^, ^30^ ^]^ Here, we investigated the effects of Rh‐GFFYE on the expressions of BRN3A and β‐III‐TUBULIN in the retina after RIR injury. The intravitreal injection of Rh‐GFFYE, saline, and control compounds was conducted one day after RIR injury. At 7 d after intravitreal injection, the number of RGCs in the RIR/Saline group (BRN3A positive) was reduced to ≈ 50% of that in the Sham group (**Figure** **5**A,C). No significant differences were observed in the number of RGCs between the Ac‐GFFYE, Nap‐GFFYE, and Saline groups. In stark contrast, treatment with Rh‐GFFYE preserved the number of RGCs to ≈ 85% of that in the Sham group. To confirm the protective effect of Rh‐GFFYE, the level of β‐III‐TUBULIN was also assessed. As expected, among the treatments, Rh‐GFFYE most strongly attenuated the RIR injury‐induced decrease in β‐III‐TUBULIN level (Figure 5B,C). In summary, the results suggest that Rh‐GFFYE can enhance the expressions of BRN3A and β‐III‐TUBULIN in the retina after RIR injury, which is of great significance for preventing the apoptosis of RGCs.

### 2.6 Rh‐GFFYE Administration Following Retinal Ischemia In Vivo Alleviates Retinal Inflammation

**Figure 6:**
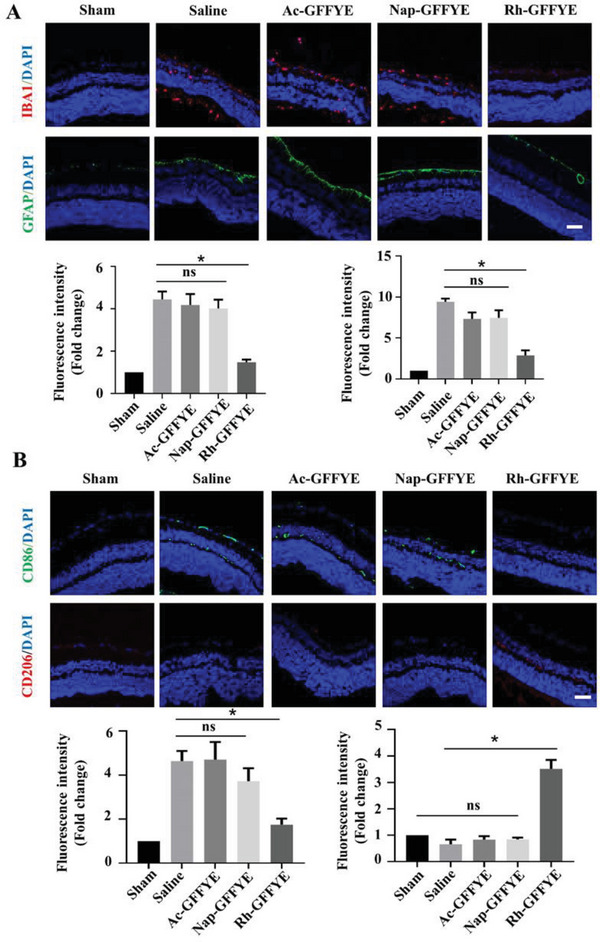
Intravitreal injection of Rh‐GFFYE inhibits the RIR injury‐induced activation of retinal gliocytes and promotes the polarization of microglia/macrophages from the M1 phenotype to the M2 phenotype. A) Representative CLSM images showing the levels of IBA1 and GFAP in retinal cryosections collected at 7 days after intravitreal injection. Fluorescence intensities of IBA1 and GFAP quantified by Image J software. Scale bar = 50 µm. *n* = 6; *p < 0.05 compared with the Saline group. B) Representative CLSM images of retinal cryosections stained for the M1 macrophage marker (CD86) and M2 macrophage marker (CD206). Fluorescence intensities of CD86 and CD206 quantified by Image J software. Scale bar = 50 µm. *n* = 6; *p < 0.05 compared with the Saline group.

In the present study, we focused on the extent of inflammation within the retinal tissue after RIR injury by evaluating the levels of an astrocyte marker (Glial fibrillary acidic protein, GFAP) and microglia marker (Ionized calcium binding adaptor molecule 1, IBA1).^[^ ^31^ ^]^ As shown in **Figure** **6**A, consistent with our hypothesis, RIR injury significantly increased the levels of IBA1 and GFAP, as reflected by an increase in fluorescence intensity. Treatment with Rh‐GFFYE clearly prevented the RIR injury‐induced upregulation of both IBA1 and GFAP. RIR injury increases the secretion of inflammatory factors including tumor necrosis factor‐α (TNF‐α) and IL‐1β.^[^ ^7^, ^29^, ^32^ ^]^ As shown in Figure S3 (Supporting Information), RIR injury increased the levels of TNF‐α and IL‐1β by ≈4 and 3 fold compared with the Sham group, respectively. In the Rh‐GFFYE group, the levels of TNF‐α and IL‐1β were only 1 and 1.3 times those in the Sham group, respectively. Based on the above results, we can preliminarily conclude that Rh‐GFFYE relieves RIR injury‐induced inflammation.

We hypothesized that the ability of Rh‐GFFYE to inhibit inflammatory response may be related to the regulation of microglia/macrophage polarization. To verify our hypothesis, we evaluated the protein levels of CD86 and CD206 in frozen retinal sections by immunofluorescence staining. Fluorescence intensity analysis indicated that RIR injury significantly increased the content of CD86 (Figure 6B), confirming that M1‐type microglia/macrophages were activated after RIR injury. However, Rh‐GFFYE induced the expression of M2‐type microglia/macrophages; as shown in Figure 6B, the level of CD206 was 3.51 times higher in the Rh‐GFFYE group than in the Saline group. Together, these findings suggest that the intravitreal injection of Rh‐GFFYE is effective at reducing inflammation associated with RIR injury and that Rh‐GFFYE treatment can inhibit M1 activation and promote microglia/macrophage reprogramming to the M2 phenotype.

### 2.7 Distribution and Safety of Rh‐GFFYE In Vivo

**Figure 7:**
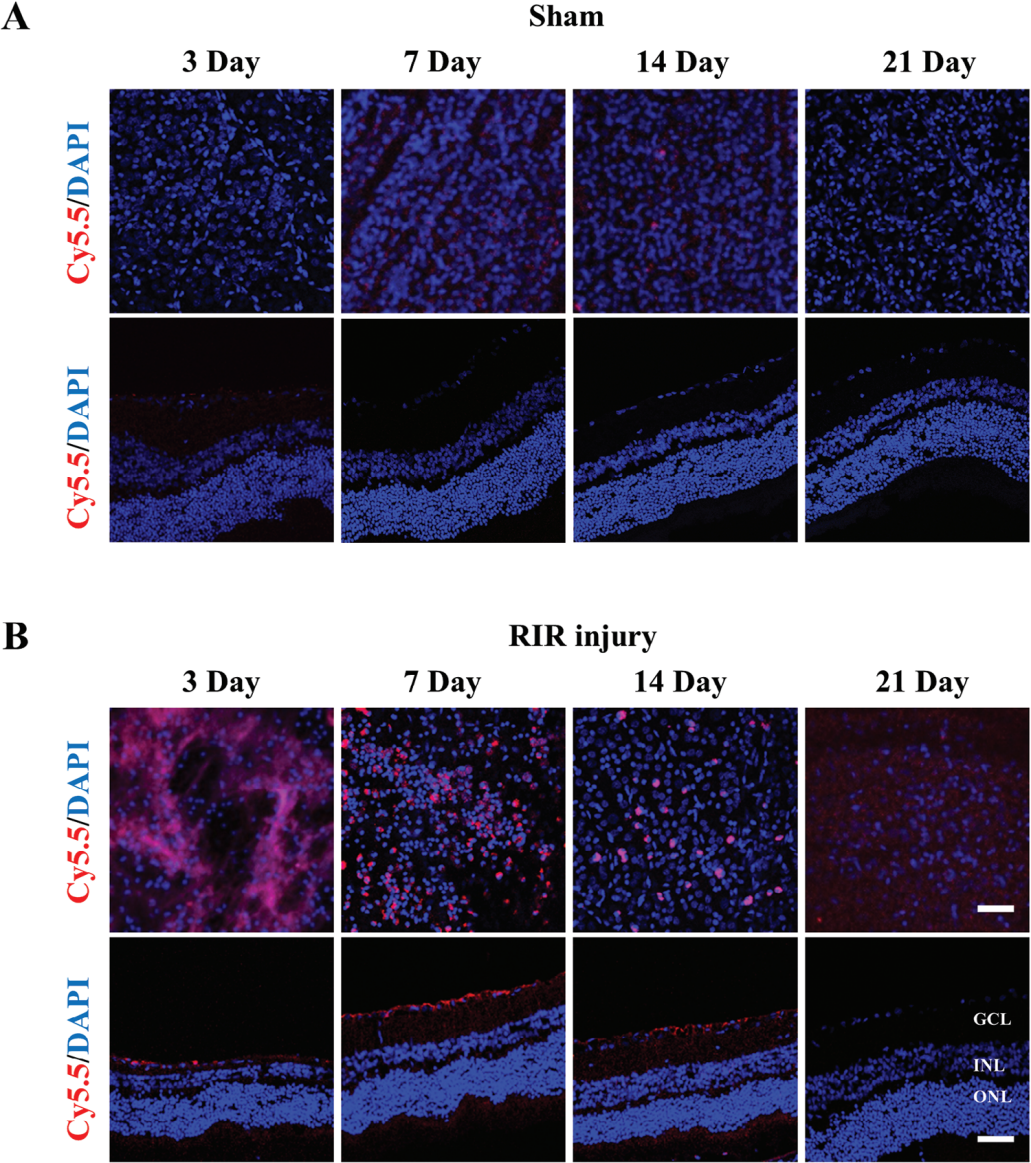
Distribution of Rh‐GFFYE in vivo. Cellular uptake of Cy5.5‐Rh‐GFFYE (red fluorescence) in retinal cryosections and retinal whole mounts observed by CLSM after intravitreal injection for 3, 7, 14, and 21 days in Sham A) and RIR injury B) group. Scale bar = 20 µm (upper); 50 µm (lower).

After observing the protective effect of Rh‐GFFYE in ischemic retinas, we evaluated the distribution of Rh‐GFFYE after intravitreal injection. Rats were divided into the Sham group and RIR injury group. Both groups received intravitreal injections of Cy5.5‐Rh‐GFFYE on the second day after RIR injury. Retinal cryosections and retinal whole flat mounts were examined at 3, 7, 14, and 21 d, and the nanofiber uptake was observed by CLSM. As shown in **Figure** **7**A,B, Cy5.5‐Rh‐GFFYE (red fluorescence) was dispersed in the retinal tissue on the third day after RIR injury, and persistent retention of Rh‐GFFYE in the retina was observed for up to 21 days after intravitreal injection. By observing the retinal cryosections, the strongest fluorescence intensity was seen in the retinal GCL on day 7, and the red fluorescence was still present on day 14. We also observed red fluorescence for up to 21 days in the retinal whole flat mounts, suggesting the sustained release of Rh‐GFFYE in the retina. Moreover, our results confirm the increased Rh‐GFFYE uptake in ischemic retina compared with the control non‐ischemic eyes. In addition, the concentration of rhein in retinas was quantified using ultra‐performance liquid chromatography/tandem mass spectrometry (UPLC‐MS/MS) at days 7 and 21 after a single intravitreal injection. As shown in Figure S4 (Supporting Information), on day 7, the concentration of rhein in the ischemic retina was 27.1 ng g^−1^, significantly higher than the concentration in the normal retina (15.2 ng g^−1^). The concentration of rhein at 21 d after intravitreal injection was approximately one‐quarter of that at day 7.

We evaluated the in vivo toxicity of Rh‐GFFYE during the treatment of RIR injury. As shown by the ERG results in Figure S5A (Supporting Information), compared with the normal group, the retinal function of the rats was not significantly changed in the Rh‐GFFYE group at 7 d after intravitreal injection. Similarly, as shown in Figure S5B (Supporting Information), there was no significant change in the morphology of the retina at 7 d after the intravitreal injection of Rh‐GFFYE. As shown in Figure S5C (Supporting Information), the serum contents of aspartate aminotransferase (AST) and alanine aminotransferase (ALT) did not show intergroup differences, indicating that liver function was within the normal range. In addition, based on the histological structures of the main visceral organs (heart, liver, spleen, lung, and kidney) in the treated and untreated groups, Rh‐GFFYE treatment did not induce noticeable histological changes (Figure S5D, Supporting Information). Taken together, these results confirm the excellent biosafety of Rh‐GFFYE and its stable release and distribution in the eye.

### 2.8 Rh‐GFFYE Regulates NF‐κB, STAT3 and PI3K/AKT/mTOR signaling pathways in RIR injury

**Figure 8:**
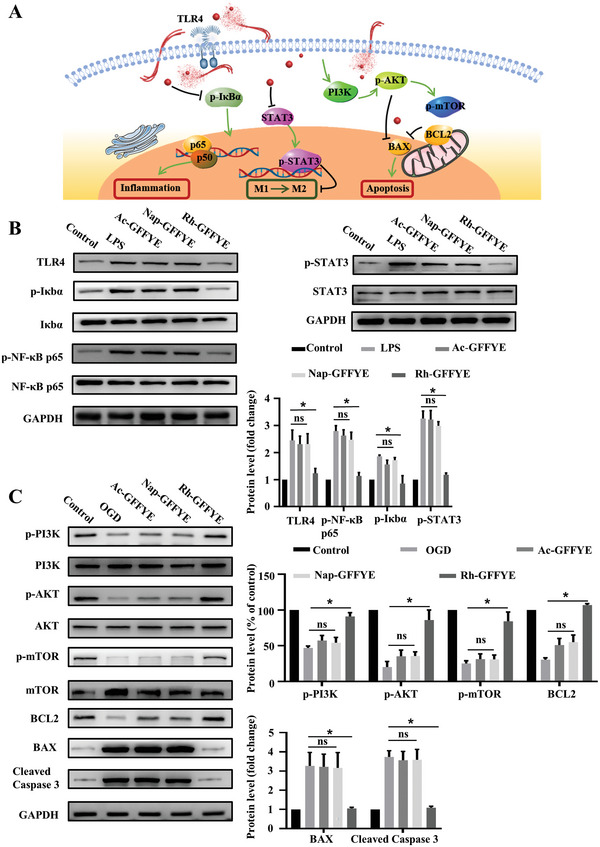
Regulation of NF‐κB, STAT3, and PI3K/AKT/mTOR signaling pathways by Rh‐GFFYE in RIR injury. A) Schematic diagram of the therapeutic mechanism of Rh‐GFFYE in RIR injury. B) Representative western blots for NF‐κB pathway proteins (TLR4, p‐NF‐κB p65, NF‐κB p65, p‐IκB, and IκB) and STAT3 pathway proteins (p‐STAT3 and STAT3) in primary macrophage with or without Rh‐GFFYE after LPS stimulation. GAPDH was used as the loading control. Bar graphs of western blot results illustrating the significant Rh‐GFFYE mediated amelioration of LPS‐induced changes in NF‐κB pathway proteins and STAT3 pathway proteins in primary macrophage treated with or without Rh‐GFFYE. The data were presented as the mean ± SD of *n* = 3; *p < 0.05 compared with the LPS group. C) Representative western blots for PI3K/AKT/mTOR pathway proteins (p‐PI3K, PI3K, p‐mTOR, mTOR, p‐AKT, and AKT) and apoptosis‐associated proteins (BCL2, BAX, and cleaved Caspase 3) in R28 cells with or without Rh‐GFFYE after OGD. GAPDH was used as the loading control. Bar graphs of western blot results illustrating the significant Rh‐GFFYE‐mediated amelioration of OGD‐induced changes in PI3K/AKT/mTOR pathway proteins and apoptosis‐associated proteins in R28 cells treated with or without Rh‐GFFYE. The data were presented as the mean ± SD of *n* = 3; *p < 0.05 compared with the OGD group.

The occurrence of RIR injury is thought to be closely related to inflammation, in which the NF‐κB signaling pathway plays a key role.^[^ ^33^ ^]^ However, the mechanism of action of rhein in RIR injury has not yet been elucidated, limiting the use of rhein in the treatment of retinal diseases. We hypothesized that Rh‐GFFYE relieves RIR injury by inhibiting the NF‐κB signaling pathway to inhibit inflammation (see **Figure** **8**A for a schematic illustration of the NF‐κB signaling pathway). We established an inflammatory model in vitro, as shown in Figure 8B, western blot analyses confirmed that LPS stimulation significantly enhanced the expressions of TLR4 and its downstream transcription factor NF‐κB compared with the Control group, consistent with a previous report.^[^ ^34^ ^]^ However, this phenomenon was reversed in the Rh‐GFFYE group, as reflected by the decreased expressions of TLR4, p‐Iκbα, and p‐NF‐κB p65 in cells. These experimental results confirm that Rh‐GFFYE indeed exerted a good anti‐inflammatory effect by inhibiting the NF‐κB signaling pathway.

Considerable evidence suggests that the STAT3 signaling pathway regulates macrophage phenotype; for instance, the activation of the M1 macrophage phenotype and its inflammatory effect are related to the phosphorylation and activation of STAT3.^[^ ^35^ ^]^ However, the ability of rhein to regulate the STAT3 signaling pathway and reprogram the macrophage phenotype in RIR injury has not been demonstrated. As shown in Figure 8B, the expression of phosphorylated STAT3 increased under LPS stimulation, indicating an increase in M1‐type macrophages; in contrast, p‐STAT3 expression decreased significantly after Rh‐GFFYE treatment, suggesting an increase in anti‐inflammatory macrophages (M2 phenotype). These results confirm that Rh‐GFFYE can reprogram macrophage polarization, which is beneficial for inhibiting inflammation and improving the retinal microenvironment.

To further confirm our hypothesis, we established an RIR injury model in rats by increasing the IOP and then intravitreally injecting saline, Rh‐GFFYE, and other drugs in the injured rats. As shown in Figure S6 (Supporting Information), immunofluorescence experiments showed that RIR injury (Saline group) significantly increased the expressions of inflammation‐related proteins, and Rh‐GFFYE treatment reversed this RIR injury‐induced increase in inflammation‐related proteins. In conclusion, our results confirm that Rh‐GFFYE inhibited the inflammatory response in vivo and in vitro by inhibiting the activation of the NF‐κB signaling pathway and the phosphorylation of downstream proteins. Meanwhile, Rh‐GFFYE can inhibit the phosphorylation of STAT3 and reprogram the polarization of macrophages; these effects play an important role in the inhibition of inflammation.

Rhein has been shown to protect myocardial cells against hypoxia/reoxygenation‐induced injury by activating the p‐AKT pathway.^[^ ^14a^ ^]^ However, to our knowledge, rhein has not been reported to regulate the PI3K/AKT/mTOR signaling pathway in RIR injury. Based on the in vivo and in vitro experimental results presented above, we hypothesized that Rh‐GFFYE can inhibit oxidative stress by activating the PI3K/AKT/mTOR signaling pathway while also reducing the expression of BAX and inhibiting the RIR injury‐induced apoptosis of RGCs. Western blot analysis showed that OGD decreased the protein expressions of p‐PI3K, p‐AKT, and p‐mTOR; these decreases in protein expression were reversed by Rh‐GFFYE, demonstrating its strong anti‐oxidative stress activity (Figure 8C). Thus, Rh‐GFFYE inhibits oxidative stress by activating the PI3K signaling pathway. Next, we surveyed the expressions of apoptosis‐related proteins in the OGD model. We found that Rh‐GFFYE increased the expressions of anti‐apoptotic protein (BCL2) and decreased the expression of the pro‐apoptotic protein BAX while also reversing the OGD‐induced increase in cleaved caspase 3 expression and inhibiting apoptosis (Figure 8C).

In vivo experiments further verified the regulatory effect of Rh‐GFFYE on the PI3K/AKT/mTOR signaling pathway. As shown in Figure S7 (Supporting Information), compared with the Control group, RIR injury (Saline group) significantly decreased the expressions of p‐PI3K, p‐AKT, and p‐mTOR, as reflected by the decrease in fluorescence intensity. Rh‐GFFYE treatment significantly reversed the RIR injury‐induced decreases in the expressions of p‐PI3K, p‐AKT, and p‐mTOR. Taken together, these results suggest that Rh‐GFFYE inhibits RIR injury‐induced oxidative stress and apoptosis in vitro and in vivo, possibly by activating the PI3K/AKT/mTOR signaling pathway and decreasing the expressions of BAX and cleaved Caspase 3.

## 3 Discussion

Previous studies have investigated the pharmacological activity of herbal‐derived natural products and shown that the oral administration of kaempferol, puerarin, and green tea extracts inhibited RIR injury and uveitis.^[^ ^36^ ^]^ In addition, the intraperitoneal administration of resveratrol was found to attenuate the loss of RGCs in RIR injury.^[^ ^37^ ^]^ However, due to the presence of the blood–retinal barrier, only ≈ 2% of the systemically administered dose eventually reaches the intraocular tissues.^[^ ^38^ ^]^ Consequently, frequent high‐dose systemic administration is required to achieve therapeutically effective concentrations, which may lead to drug‐related toxicity. Currently, intravitreal injection is still considered the best delivery strategy for ophthalmic therapeutics when treating posterior segment eye diseases. Therefore, sustained‐release drug delivery systems are needed to avoid complications caused by multiple injections of drugs with short half‐lives.^[^ ^39^ ^]^ Self‐assembling peptides can generate different structures including hydrogels and nanofibers, which allow for efficient encapsulation and drug conjugation.^[^ ^18b^ ^]^ In addition, peptides have excellent biocompatibility and permeability in the vitreous compared to other supramolecular materials. Peptides based on diphenylalanine peptide (FF) have proven to be excellent self‐assembled molecules that can form nanostructures through π–π stacking.^[^ ^40^ ^]^ In addition, we previously demonstrated that Rh‐GFFYERGD can form nanofibers.^[^ ^19^ ^]^ This study provides new results on the modulation of the retinal microenvironment by peptide‐based rhein nanofibers (Rh‐GFFYE) in RIR injury with relevance to the treatment of retinal ischemic diseases and glaucoma.

In our study, Rh‐GFFYE improved cell viability, as evidenced by reduced LDH release and increased ATP levels in cells subjected to OGD. These results are consistent with previous findings in traumatic brain injury as well as myocardial hypoxia/reoxygenation injury.^[^ ^14^, ^41^ ^]^ The accumulation of ROS is one of the reasons for the decline of cell viability.^[^ ^42^ ^]^ In our previous study, we also found that RIR injury can cause excessive production of ROS,^[^ ^29^ ^]^ and Rh‐GFFYE nanofibers can reduce the excessive production of ROS in this work, which is consistent with several previous studies that rhein can inhibit ROS overproduction;^[^ ^43^ ^]^ however, to the best of our knowledge, rhein was first reported to reduce ROS in a RIR injury model. In addition, we found that Rh‐GFFYE can stabilize the mitochondrial membrane potential, which can reflect the function of mitochondria as the site of ROS production.^[^ ^22^, ^44^ ^]^ Thus, these results suggest that Rh‐GFFYE nanofibers suppress oxidative stress, which is an early event in RIR injury.

RIR injury is mainly manifested by decreased retina thickness, loss of RGCs, and neuroinflammation, leading to visual impairment.^[^ ^7^, ^45^ ^]^ Rhein have shown therapeutic effects in various inflammatory diseases, including ischemia‐reperfusion injury in the brain and heart along with pneumonia and ulcerative colitis.^[^ ^14^, ^46^ ^]^ Our results demonstrated that Rh‐GFFYE nanofibers exert retinal protective effects by reducing oxidative stress, neuroinflammation, and retinal cell apoptosis. These results provide a fundamental insight into the mechanism behind the action of Rh‐GFFYE in the retina under ischemic injury. Oxidative stress can cause inflammation, which is the main event in the change of retinal microenvironment in RIR injury.^[^ ^47^ ^]^ Glial cells such as astrocytes, microglia, and Müller cells are activated during RIR injury; these activated glial cells change the retinal microenvironment, eventually leading to the loss of RGCs.^[^ ^48^ ^]^ Han et al. demonstrated that ribonuclease can reduce the levels of GFAP proteins and inhibit inflammatory factors such as TNF‐α to reduce RIR injury in mice.^[^ ^49^ ^]^ Our in vivo data demonstrate that Rh‐GFFYE can effectively inhibit the expressions of GFAP and IBA1 and reduce inflammation, which is consistent with the in vitro data. The process of inflammation is also usually related to the polarization of macrophages.^[^ ^50^ ^]^ In this work, Rh‐GFFYE reprogramed macrophages into the anti‐inflammatory M2 phenotype, which is supported by the previous research that rhein was found to reprogram the macrophage phenotype in an arthritis model.^[^ ^24b^ ^]^

Prior to this study, few studies investigated the uptake and distribution of rhein‐based nanomaterials not only in the normal retina but also in the ischemic retina. To our knowledge, the retinal toxicity of rhein has been determined in only a limited manner. After 21 days of single intravitreal injection of Rh‐GFFYE in an in vivo model, we could detect Cy5.5 labeled to the nanofibers using the CLSM. In parallel, we found a time‐dependent distribution of monomeric rhein in the retina, indicating that Rh‐GFFYE can release rhein in a sustained manner. Taken together, our results indicate Rh‐GFFYE does not cause retinal cytotoxicity, any deleterious effects on ERG function, as well as increases in inflammatory markers.

The inflammatory response caused by RIR injury is closely related to the activation of the NF‐κB signaling pathway.^[^ ^49^ ^]^ Rhein has been shown to inhibit neuroinflammation, primarily through the inhibition of the NF‐κB signaling pathway.^[^ ^17^ ^]^ In the current study, we found OGD induced the activation of the NF‐κB signaling pathway in vitro, and Rh‐GFFYE inhibited the phosphorylation of inflammation‐associated proteins and reduced inflammatory responses. Li et al. reported that the NF‐κB‐STAT3 signaling pathway was activated in an oxygen‐induced retinopathy model, resulting in the upregulation of M1‐type microglia/macrophages and the release of inflammatory factors such as TNF‐α.^[^ ^51^ ^]^ Our previous study reported that STAT3 may be associated with the polarization of microglia in RIR injury and that rhein can inhibit the activation of the STAT3 signaling pathway; however, the ability of rhein to regulate the STAT3 signaling pathway in RIR injury has not been reported.^[^ ^9^, ^24^, ^52^ ^]^ Our in vitro and in vivo results show that Rh‐GFFYE can inhibit the phosphorylation of STAT3 protein, which is similar to our previous report on the mechanism of action of ginsenoside Rg3 liposomes in RIR injury.^[^ ^9a^ ^]^ These results provide a rationale for the regulation of microglia polarization by the natural product rhein.

The PI3K/AKT/mTOR signaling pathway is the core site of oxidative stress, inflammation, and apoptosis, and it is closely related to the occurrence of RIR injury.^[^ ^53^ ^]^ Aqueous extract of turnip reversed the OGD‐induced decrease in p‐PI3K level and exerted a neuroprotective effect in cerebral ischemia‐reperfusion injury.^[^ ^54^ ^]^ Our study shows that Rh‐GFFYE can inhibit the activation of PI3K/AKT/mTOR signaling pathway, which is consistent with the previously reported mechanism of action of natural products resveratrol and ligustrazine on RIR injury inhibition.^[^ ^37^, ^55^ ^]^ Nevertheless, to the best of our knowledge, this is the first report that rhein regulates the PI3K/AKT/mTOR signaling pathway in retinal diseases. Furthermore, the expression of BAX was inhibited by Rh‐GFFYE, whereas the expression of BCL2 was increased by Rh‐GFFYE treatment. These results suggest that Rh‐GFFYE inhibits RIR injury‐induced retinal cell apoptosis, which may be the one of reasons for its attenuation of RIR.

## 4 Conclusion

In this study, we 1) developed peptide‐based nanofibers that accumulate in the retina and are safely and easily taken up by retinal cells; 2) applied this nanoplatform for rhein delivery in an in vitro model of OGD and an in vivo model of RIR injury; 3) demonstrated that Rh‐GFFYE can inhibit oxidative stress by upregulating the PI3K/AKT/mTOR signaling pathway; and 4) demonstrated that Rh‐GFFYE can downregulate the NF‐κB and STAT3 signaling pathways, thereby reversing the pro‐inflammatory microenvironment of the retina by inhibiting the activation of M1 microglia and promoting the transformation of the M1 phenotype to the M2 phenotype. Further development and optimization of this unique drug delivery nanoplatform may lead to a viable natural product‐ and peptide‐based nano‐delivery approach for the treatment of RIR injury‐associated eye diseases.

## 5 Experimental Section

### Chemicals and Reagents

Rhein standard (purity > 98%) was purchased from Sangon Biotech (Shanghai, China). Fmoc‐amino acids were obtained from GL Biochem (Shanghai, China). All other materials used to synthesis Rh‐GFFYE and the other two compounds were obtained from Alfa (Beijing, China). Dulbecco's modified eagle medium (DMEM), penicillin/streptomycin were obtained from Life Technologies (Carlsbad, CA, USA). Calf serum was purchased from FuHeng BioLogy (Shanghai, China).H_2_DCF‐DA was purchased from Beyotime Biotechnology Co., Ltd. (Shanghai, China). 4′,6‐Diamidino‐2‐phenylindole (DAPI) was purchased from Amy Jet Scientific Inc Co., Ltd. (Wuhan, China).

### Preparation of Peptide Derivatives

The peptide derivatives Rh‐GFFYE, Ac‐GFFYE, and Nap‐GFFYE were prepared similarly to our previous study.^[^ ^19^ ^]^ Briefly, they were synthesized by standard Fmoc solid‐phase peptide synthesis (SPPS), which used 2‐chlorotrityl chloride resin and the corresponding N‐Fmoc protected amino acids with side chains suitably protected by a *tert‐*butyl group or *tert‐*butyloxycarbonyl group. The resulting crude products were purified by high‐performance liquid chromatography (HPLC, LUMTECH, German) and lyophilization. These compounds were characterized by ^1^H‐NMR (Bruker ARX 500) and MS. MS was conducted at the Agilent InfinityLab LC/MSD system.

### Preparation and Characterization of Nanofibers

The assembly of the nanofibers was achieved using a heating‐cooling process. Briefly, 1 mg of Rh‐GFFYE was dissolved in 250 µL PBS and the pH was adjusted to 7.4 using sodium carbonate. The suspension was ultrasonically treated for 15 min and was heated to ≈70 °C and then cooled back to room temperature. The microstructure of the nanofibers was observed with TEM (HITACHI HT7700, Japan). The acceleration voltage is 100 kV. Micropipet was used to place hydrogels containing 0.2 wt. % of Rh‐GFFYE onto carbon‐coated copper grids. Nanofibers were stained with uranyl acetate for 1 min, and then the excess dye was absorbed with filter paper and the sample was dried overnight in a dryer.

### Cell Culture and Treatment with Rh‐GFFYE

The retinal precursor cell line R28 was purchased from Kerafast (Boston, MA, USA) and cultured according to the procedures recommended by the supplier. Briefly, the cells were grown and maintained in DMEM complete medium containing 420 mL DMEM, 15 mL sodium bicarbonate (Sigma‐Aldrich, St. Louis, MO, USA), 50 mL calf serum, 5 mL MEM non‐essential amino acids, 5 mL l‐glutamine (GIBCO, Grand Island, NY, USA), and 0.625 mL Gentamicin (80 mg mL^−1^; Solarbio Life Sciences Co., Ltd, Beijing, China) at 37 °C in a humidified atmosphere containing 5% CO_2_. The stock solutions of Rh‐GFFYE and control compounds were diluted to varying concentrations with DMEM complete medium just before cell treatment.

### Cellular Uptake

R28 cells were used to investigate the cellular internalization of Rh‐GFFYE. Briefly, cells were seeded in 35‐mm glass‐bottom Petri dishes (1 × 10^5^ mL^−1^ cells) and cultured overnight. Meanwhile, Cy5.5‐GFFYE was dispersed in PBS at a final concentration of 8 mM. Afterward, Cy5.5‐GFFYE suspension and Rh‐GFFYE nanofibers were vortex mixed at a molar ratio of 1:8 and left overnight at room temperature in the dark to self‐assemble into Cy5.5‐Rh‐GFFYE. Cy5.5‐Rh‐GFFYE was then incubated with R28 cells at a concentration of 20 µM for various times (4, 8, and 24 h), and the cells were observed by CLSM (ZEISS LSM 800, Germany).

### In Vitro OGD Model and Detection of Cell Activity

An in vitro OGD model was constructed based on the method detailed by Ryou et al.^[^ ^56^ ^]^ Briefly, R28 cells were cultured in a glucose‐free medium and subjected to hypoxia (1% O_2_, 5% CO_2_) for 24 h. The cells were then re‐oxygenated (21% O_2_, 5% CO_2_) for 6 h to simulate RIR. The ATP levels were measured using a CellTiter‐Lumi Plus luminescent cell viability assay (Beyotime, Beijing, China). The cell membrane integrity of the R28 cells was evaluated by LDH release assay (Beyotime, Beijing, China).

### Measurement of Intracellular ROS

R28 cells were seeded at a density of 1 × 10^5^ mL^−1^ cells in 35‐mm glass‐bottom Petri dishes and incubated overnight. Before OGD injury, the cells were treated with Rh‐GFFYE, rhein, Nap‐GFFYE, or Ac‐GFFYE at a concentration of 20 µM for 24 h. The OGD‐injured cells were then assayed for intracellular ROS production by H_2_DCF‐DA staining. Briefly, the cells were treated with 10 µM H_2_DCF‐DA for 30 min in the cell culture incubator. To remove the unincorporated dye, the cells were washed with PBS and the oxidation of H_2_DCF‐DA was detected by CLSM.

### Detection of Mitochondrial Membrane Potential

A mitochondrial membrane potential assay kit with JC‐1 (Beyotime, Beijing, China) was used to monitor the changes in the mitochondrial membrane potential of R28 cells subjected to OGD following the procedure recommended by the supplier. Briefly, R28 cells were seeded in dishes at a density of 1 × 10^5^ mL^−1^ cells and cultured overnight. The cells were treated with Rh‐GFFYE, rhein, Nap‐GFFYE, or Ac‐GFFYE at a concentration of 20 µM for 24 h prior to OGD. Following treatment and recovery, the cells were washed three times with PBS and then incubated with 20 µM JC‐1 for 20 min at 37 °C. After removing the JC‐1 staining solution, the cells were washed three times with JC‐1 dyeing buffer, and 2 mL DMEM was added for CLSM imaging.

### Extraction and Culture of Mouse BMDMs

The isolation of mouse BMDMs was performed according to the method of Pineda‐Torra et al.^[^ ^57^ ^]^ Briefly, the femurs and tibias of C57BL/6 mice were carefully separated and transferred into PBS containing 10% penicillin/streptomycin. The BMDMs were flushed out with a syringe and filtered through a 70‐µm strainer. To further purify the BMDMs, lysis buffer was added to remove the red blood cells. The BMDMs were cultured in a DMEM medium containing macrophage colony‐stimulating factor (MCE, Monmouth Junction, NJ, USA).

### Measurement of Cytokines

BMDMs (1 × 10^5^ mL^−1^ cells in a volume of 1 mL per well in a 24‐well plate) were pretreated with Rh‐GFFYE, rhein, Nap‐GFFYE, and Ac‐GFFYE at a concentration of 20 µM for 1 h prior to the addition of LPS (100 ng mL^−1^) for the specified times (6, 24, and 48 h). The supernatants were collected, and the concentrations of IL‐1β and IL‐6 in the culture medium were measured using ELISA kits (Beyotime, Beijing, China) according to the manufacturer's instructions.

### Animals

Eight‐week‐old male Sprague‐Dawley rats (200–250 g) were purchased from Pengyue Experimental Animal Company (Jinan, China). The Yantai University Committee for the Care and Use of Laboratory Animals authorized the animal care and experimental procedures, which followed the National Institutes of Health Guide for the Care and Use of Laboratory Animals in Research (Permit Number: YTU20210901). All rats were housed under a 12 h dark/light cycle and given unlimited access to water and food. The rats were anesthetized with intraperitoneal injections of ketamine (100 mg kg^−1^) and xylazine (7 mg kg^−1^) prior to intravitreal injection

### Rat Model of RIR Injury and Intravitreal Injection

The RIR injury model was established as previously described.^[^ ^29^ ^]^ Prior to establishing the model, each anesthetized rat was administered with 0.3% ofloxacin eye drops. Topyramide eye solution was used to dilate the pupil, and proparacaine hydrochloride was used as an eye anesthetic. A 30‐gauge needle containing a balanced salt solution was cannulated into the anterior chamber. The infusion bottle was raised to a vertical distance of 150 cm, and the IOP of the rat was found to be ≈70 mmHg using a pressure flush tonometer. The sham operation, which served as the control, was performed without elevating the IOP. After 60 min, the needle was withdrawn, the IOP was normal, the posterior segment was pale, and the blood supply was restored, confirming the successful establishment of the RIR injury model. Erythromycin eye ointment was used to prevent bacterial infection. During the experiment, each animal's body temperature was kept between 36.5 and 37°C.

At 24 h after establishing the RIR injury model, the rats were randomly divided into five groups: Sham, Saline, Rh‐GFFYE, Ac‐GFFYE, and Nap‐GFFYE. The methods for anesthesia and pupil dilation were the same as described above. Intravitreal injection was accomplished using a Hamilton syringe, and 4 µL of Rh‐GFFYE was injected to obtain a final rhein concentration of 20 µM in the vitreous humor.^[^ ^58^ ^]^

### Hematoxylin and Eosin (H&E) Staining of the Retina

The changes in retinal morphology caused by RIR injury were visualized by H&E staining. At 7 days after the intravitreal injection of Rh‐GFFYE, the eyeballs were removed and then fixed in 4% paraformaldehyde. The eye tissue was then embedded in paraffin, and 4‐µm‐thick slices were prepared. The H&E staining procedure described previously was followed.^[^ ^59^ ^]^

### TUNEL Assay

The One‐step TUNEL Apoptosis Assay Kit (Beyotime, Beijing, China) was used to perform the TUNEL assay. Briefly, cryosections were washed by PBS for three times and then incubated with 0.3% Triton X‐100 at room temperature. TUNEL test solution was prepared to stain the cryosections, after rinsing three times with PBS, DAPI was added to counterstain each cryosection sample. Sealed with a cover glass, the slides were then observed by CLSM.

### Evaluation of Retinal Function

Before ERG recording, all the rats were randomly divided into five groups (Sham, Saline, Nap‐GFFYE, Ac‐GFFYE, and Rh‐GFFYE) and adapted in the dark for 12 h. All ERG procedures were performed in a dark room under dim redlight illumination according to a previous report.^[^ ^60^ ^]^ The rats were anesthetized with 1% pentobarbital and 10% isoflurane. Topicaramide eye solution was applied topically to dilate the pupils, and 1% sodium hyaluronate was used for lubrication and hydration. The skin and tail between the ears were inserted with stainless‐steel electrodes to serve as reference and ground leads, respectively. ERG was conducted using a gold‐plated wire loop contacting the surface of the cornea as the active electrode. For scotopic ERG, the responses to white flashes of 3.00 cd∙s∙m^−2^ were recorded. The flash recordings were obtained synchronously from both eyes, and each recording was averaged three times. The animals were placed on a thermal platform that was kept at 37 °C throughout the experiment.

### Evaluation of Retinal Mitochondria

The dissected retina was fixed in 2.5% phosphate glutaraldehyde overnight. Samples were observed by TEM after being post‐fixed, embedded, cut, and mounted.

### Preparation of Frozen Retina Sections and Immunofluorescent Staining of the Retina

The RIR‐injured rats were euthanized at 7 days after the intravitreal injection of Rh‐GFFYE. Immediately after removal, the eyeball was fixed in 4% paraformaldehyde for 2 h at 4 °C, dehydrated overnight in 30% sucrose solution, embedded in an optimal cutting temperature compound embedding bottom box (17 × 17 × 5 mm), and stored at −80 °C. Slices with thicknesses of 8 µm were created using a frozen slicer at −22 °C. The slices were placed on adhesive slides and dried at room temperature for >30 min before proceeding to the next step. For immunofluorescent staining, the retinal sections were permeabilized with enhanced immunostaining permeabilization buffer (Beyotime, Beijing, China) for 20 min, blocked with blocking buffer (Beyotime, Beijing, China), and incubated overnight at 4 °C with the following primary antibodies: anti‐β‐III‐TUBULIN, anti‐IBA1, anti‐GFAP, anti‐BRN3A (Abcam, UK), anti‐CD86, and anti‐CD206 (Cell Signaling Technology, Danvers, MA, USA). After rinsing three times with PBS, the sections were incubated with secondary antibodies at room temperature for 2 h, rinsed three times with PBS, and counterstained with DAPI. Sealed with a cover glass, the slides were then observed by CLSM.

### In Vivo Retinal Distribution Study

To evaluate the distribution of Rh‐GFFYE in RIR‐injured and normal retinas, rats were divided into two groups. At 3, 7, 14, and 21 days after a single intravitreal injection of Cy5.5‐Rh‐GFFYE, the rats were killed and retinas were obtained. Retinal cryosections and retinal whole flat mounts were examined by CLSM to observe the distribution of Rh‐GFFYE in vivo.

To further evaluate the concentration of rhein in retinas, the retinas were collected at 7 and 21 days after a single intravitreal injection of Rh‐GFFYE. The retinas were homogenized using a cryogenic homogenizer (ServiceBio, Wuhan, China), and the supernatants were collected after centrifugation for further analysis. The samples were suspended in ethyl acetate and centrifuged for 5 min to obtain the supernatants which were then dried under nitrogen gas flow. The residues were dissolved with mobile phase solution and then quantified using the UPLC‐MS/MS (AB Sciex Triple Quad TM 4500 system, MA, USA) assay.

### Safety Evaluation

For safety evaluation, rats were intravitreally injected Rh‐GFFYE nanofibers. 7 days after injection, ERG was used to detect the retinal function and H&E staining was used to detect the morphology of retina. Orbital blood was taken to detect the contents of ALT and AST in plasma. The major organs (heart, liver, spleen, lung, kidney) were harvested for H&E staining to observe changes in each major organ.

### Western Blot Analysis

The protein concentrations were determined using a BCA protein assay kit (Beyotime, Beijing, China). Proteins were electrophoresed on 10% SDS‐polyacrylamide electrophoresis gels and transferred onto a PVDF membrane (Millipore, Merck, Germany). The membranes were blocked in 5% skim milk for 4 h at room temperature. The following were used as primary antibodies: anti‐TLR4 (Santa Cruz Biotechnology, Dallas, TX, USA), anti‐p‐NF‐κB p65, anti‐p‐PI3K, anti‐STAT3, anti‐p‐STAT3 (Beyotime, Beijing, China), anti‐NF‐κB p65, anti‐p‐IκBα, anti‐IκBα, anti‐p‐mTOR, anti‐mTOR, anti‐p‐AKT, anti‐AKT (Cell Signaling Technology, Danvers, MA, USA), anti‐PI3K, anti‐cleaved Caspase 3, anti‐BAX, and anti‐BCL2 (Proteintech, Wuhan, China). Glyceraldehyde‐3‐phosphate dehydrogenase (GAPDH; Proteintech, Wuhan, China) served as the loading control for total proteins. The blots were incubated with the primary antibodies overnight at 4 °C. After washing three times with TBST, the blots were incubated with anti‐mouse secondary antibodies and anti‐rabbit secondary antibodies (Beyotime, Beijing, China) for 1 h at room temperature. Western blots were performed for three independent experiments of each treatment. The intensity of each band was quantified with Image J software (Version 1.52p, NIH).

### Statistical Analysis

Graph Pad Prism 9 (La Jolla, USA) was used to analyze the data, and all data were presented as the mean ± standard deviation (SD). The sample size (*n*) for each statistical analysis had been reported in the corresponding “Figure legends.” Treatment‐related differences were evaluated by one‐way analysis of variance (ANOVA) followed by Dunnett's test (for comparisons between different concentrations and the vehicle control) or two‐way ANOVA followed by Tukey's multiple comparison test (for comparisons between different treatment groups). A difference was considered statistically significant when the *p*‐value was <0.05.

## Conflict of Interest

The authors declare no conflict of interest.

## Supporting information

Supporting Information

## Data Availability

The data that support the findings of this study are available from the corresponding author upon reasonable request.
