# Supplementary material for: Retinal Microenvironment‐Protected Rhein‐GFFYE Nanofibers Attenuate Retinal Ischemia‐Reperfusion Injury via Inhibiting Oxidative Stress and Regulating Microglial/Macrophage M1/M2 Polarization
Source: Adv Sci (Weinh). 2023 Aug 31;10(30):2302909. doi: 10.1002/advs.202302909 (PMC10602545; doi:10.1002/advs.202302909)
Supplement: Supplementary file 1 — Supporting Information [file ADVS-10-2302909-s001.pdf]

ADVANCED SCIENCE
Open Access

Supporting Information

for *Adv. Sci.*, DOI 10.1002/advs.202302909

Retinal Microenvironment-Protected Rhein-GFFYE Nanofibers Attenuate Retinal Ischemia-Reperfusion Injury via Inhibiting Oxidative Stress and Regulating Microglial/Macrophage M1/M2 Polarization

Zhuhong Zhang, Shengjun Peng, Tengyan Xu, Jia Liu, Laien Zhao, Hui Xu, Wen Zhang, Yuanying Zhu and Zhimou Yang**

Retinal microenvironment-protected rhein-GFFYE nanofibers attenuate retinal ischemia-reperfusion injury via inhibiting oxidative stress and regulating microglial/macrophage M1/M2 polarization

Zhuhong Zhang, Shengjun Peng, Tengyan Xu, Jia Liu, Laien Zhao, Hui Xu, Wen Zhang, Yuanying Zhu, and Zhimou Yang**

A

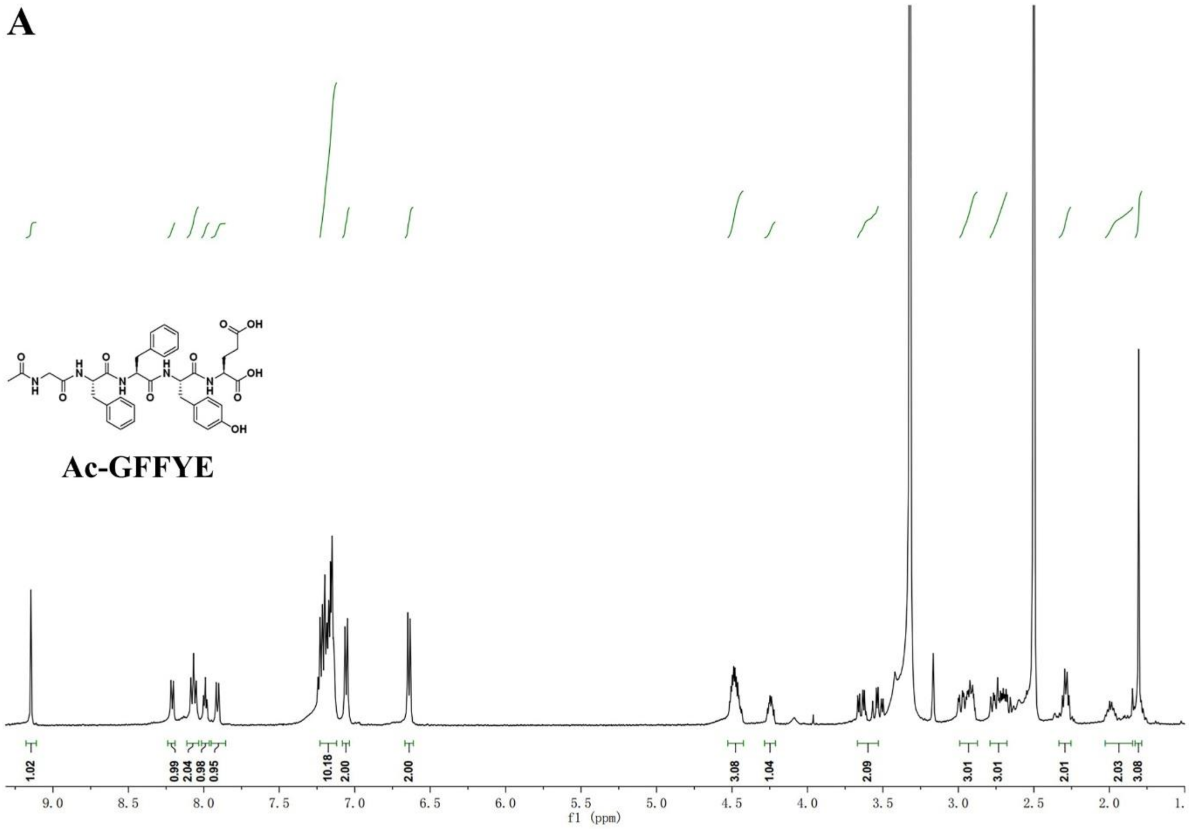

B

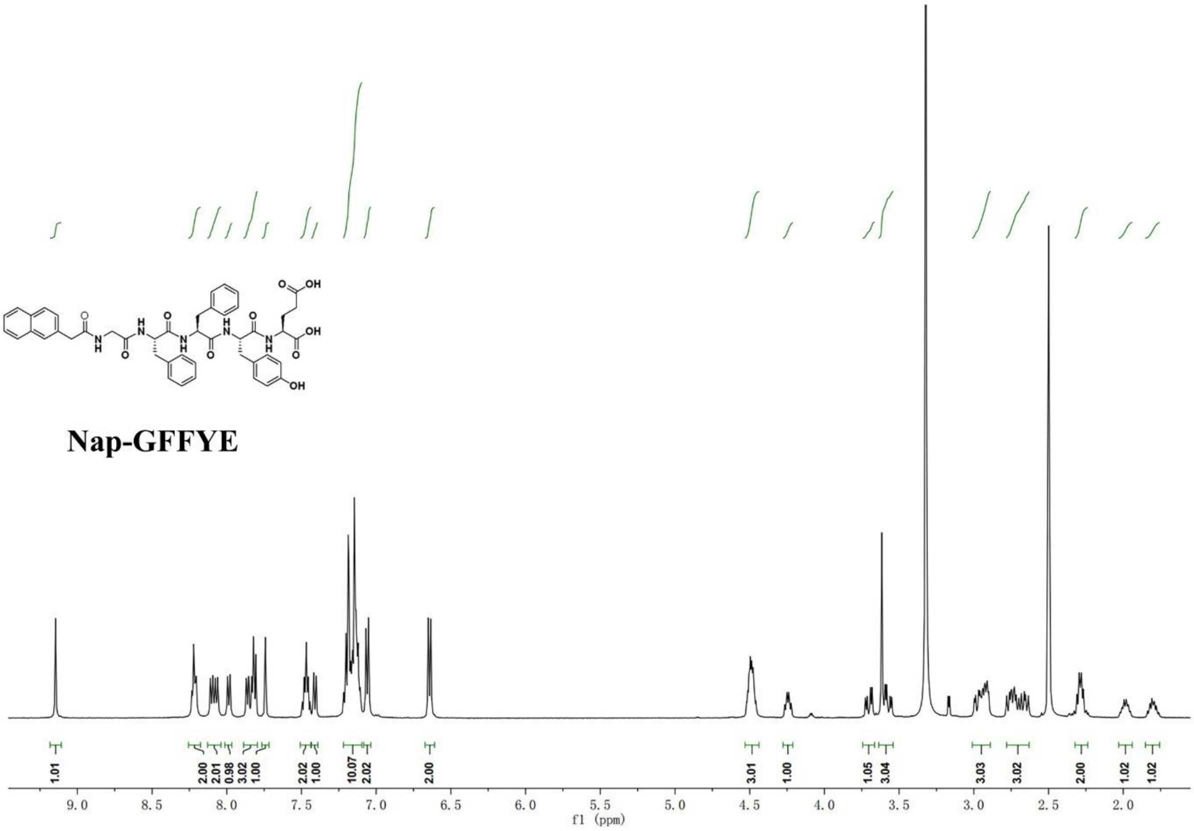

C

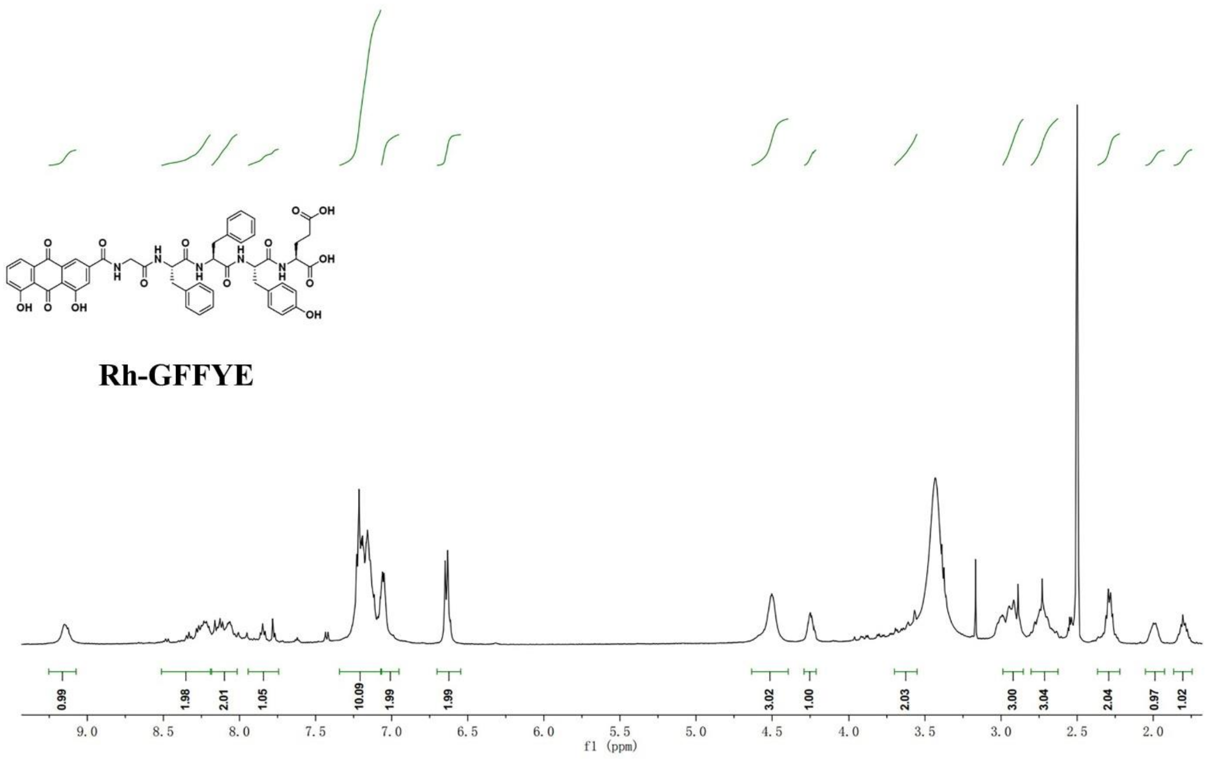

Figure S1. ^{1}H NMR spectrum of three compounds. A) ^{1}H NMR (500 MHz, DMSO-d_6) spectrum of Ac-GFFYE. δ 9.15 (s, 1H), 8.21 (d, *J* = 7.8 Hz, 1H), 8.07 (t, *J* = 8.8 Hz, 2H), 7.99 (t, *J* = 5.7 Hz, 1H), 7.91 (d, *J* = 8.4 Hz, 1H), 7.23 – 7.12 (m, 10H), 7.06 (d, *J* = 8.3 Hz, 2H), 6.64 (d, *J* = 8.1 Hz, 2H), 4.48 (dtt, *J* = 13.1, 8.7, 4.3 Hz, 3H), 4.25 (s, 1H), 3.67 – 3.53 (m, 2H), 2.99 – 2.87 (m, 3H), 2.79 – 2.68 (m, 3H), 2.33 – 2.25 (m, 2H), 2.03 – 1.85 (m, 2H), 1.81 (s, 3H). B) ^{1}H NMR (500 MHz, DMSO-d_6) spectrum of Nap-GFFYE. δ 9.15 (s, 1H), 8.22 (q, *J* = 4.7, 3.5 Hz, 2H), 8.09 (dd, *J* = 16.2, 8.1 Hz, 2H), 7.99 (d, *J* = 8.3 Hz, 1H), 7.89 – 7.80 (m, 3H), 7.74 (s, 1H), 7.47 (tt, *J* = 7.0, 5.2 Hz, 2H), 7.41 (dd, *J* = 8.4, 1.7 Hz, 1H), 7.22 – 7.10 (m, 10H), 7.06 (d, *J* = 8.2 Hz, 2H), 6.64 (d, *J* = 8.3 Hz, 2H), 4.49 (qd, *J* = 9.7, 9.2, 4.1 Hz, 3H), 4.24 (td, *J* = 8.3, 5.1 Hz, 1H), 3.70 (dd, *J* = 16.7, 5.7 Hz, 1H), 3.64 – 3.54 (m, 3H), 3.01 – 2.89 (m, 3H), 2.78 – 2.63 (m, 3H), 2.32 – 2.24 (m, 2H), 1.99 (dq, *J* = 13.7, 7.5, 6.8 Hz, 1H), 1.85 – 1.76 (m, 1H). C) ^{1}H NMR (500 MHz, DMSO-d_6) spectrum of Rh-GFFYE. δ 9.15 (s, 1H), 8.24 (td, *J* = 16.0, 15.6, 7.8 Hz, 2H), 8.18 – 8.02 (m, 2H), 7.94 – 7.74 (m, 1H), 7.34 – 7.07 (m, 10H), 7.05 (q, *J* = 6.6, 4.6 Hz, 2H), 6.63 (t, *J* = 8.8 Hz, 2H), 4.63 – 4.39 (m, 3H), 4.25 (tt, *J* = 9.0, 4.8 Hz, 1H), 3.70 – 3.55 (m, 2H), 2.99 – 2.85 (m, 3H), 2.80 – 2.63 (m, 3H), 2.37 – 2.22 (m, 2H), 1.99 (s, 1H), 1.81 (tt, *J* = 10.4, 6.1 Hz, 1H).

A

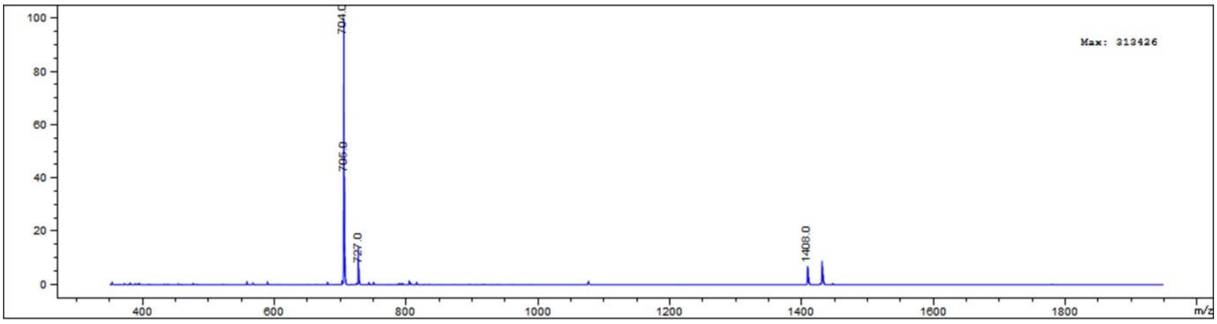

B **Ac-GFFYE**

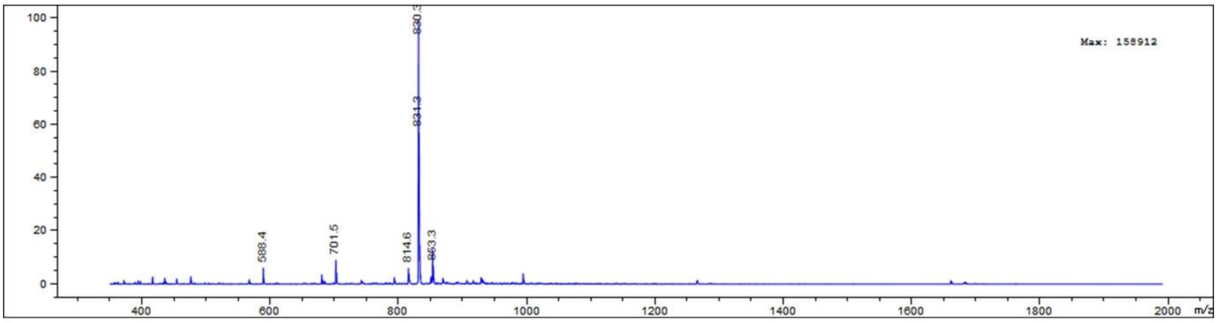

C **Nap-GFFYE**

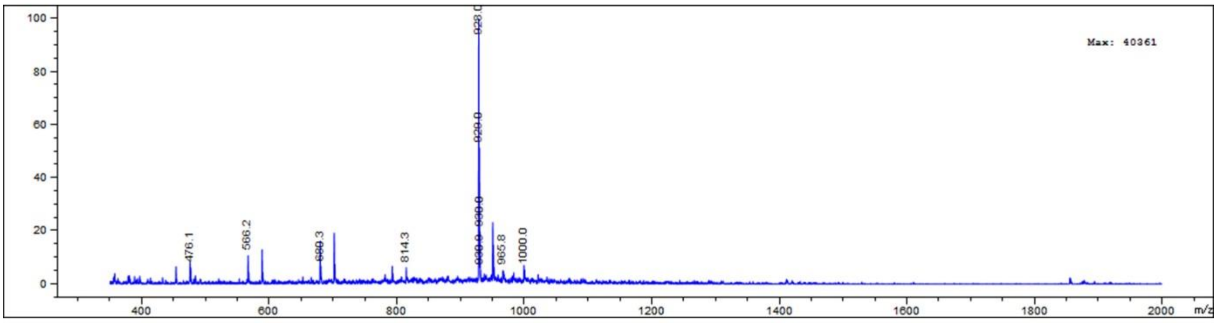

Rh-GFFYE

Figure S2. Mass spectrometry (MS) spectrum of three compounds. A) MS spectrum of Ac-GFFYE. MS: calcd. M = 703.7, obsvd. $(M+H)^+$ = 704. B) MS spectrum of Nap-GFFYE. MS: calcd. M = 829.9, obsvd. $(M+H)^+$ = 830.3 C) MS spectrum of Rh-GFFYE. MS: calcd. M = 927.9, obsvd. $(M+H)^+$ = 928.0.

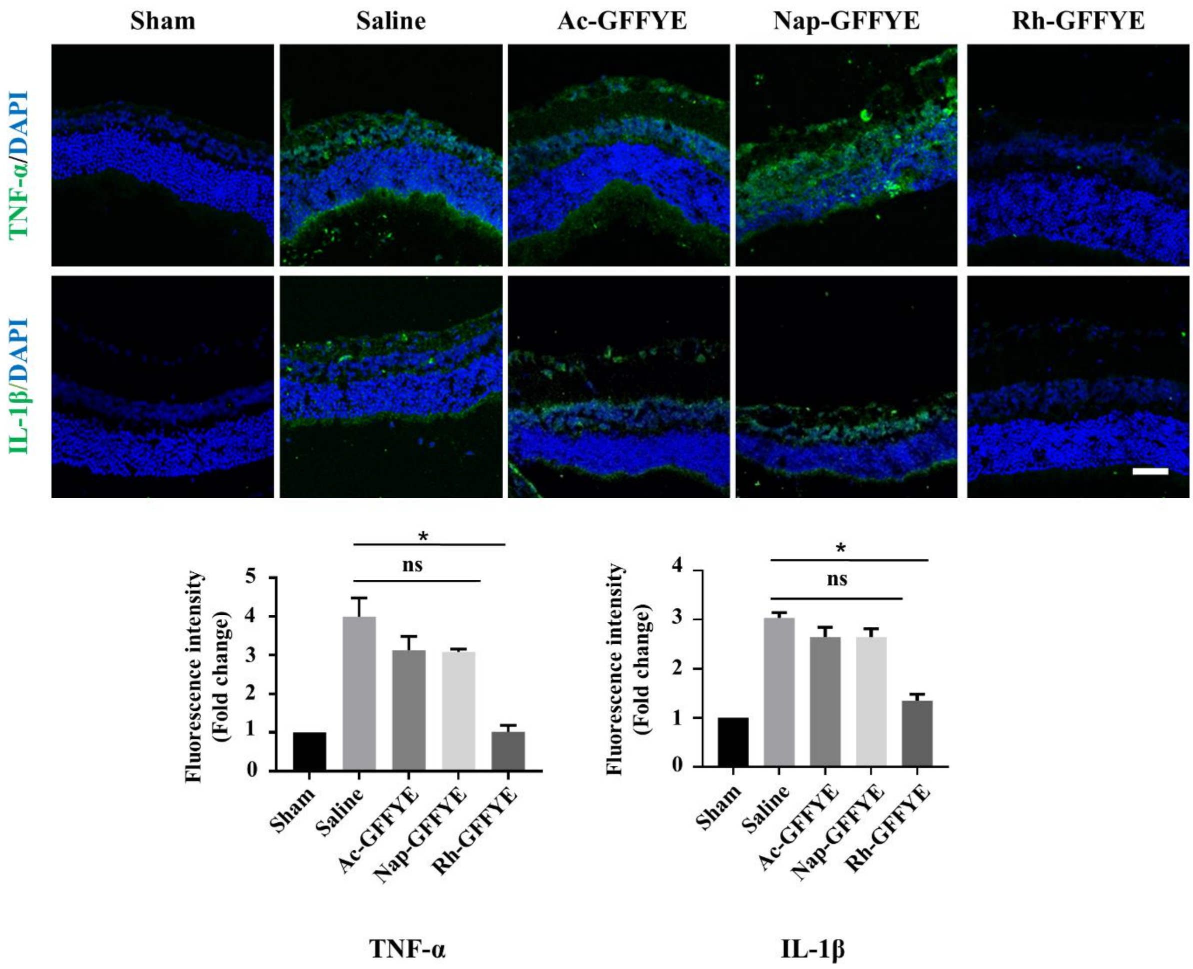

Figure S3. Inhibition of RIR injury-induced secretion of inflammatory cytokines by Rh-GFFYE following intravitreal injection. Representative CLSM images showing the levels of inflammatory factor TNF-α and IL-1β in retinal cryosections collected at 7 days after intravitreal injection. Fluorescence intensities of TNF-α and IL-1β quantified by Image J software. Scale bar = 50 μm. The data were presented as the mean ± SD of $n = 6$; $^{*}P < 0.05$ compared with the Saline group.

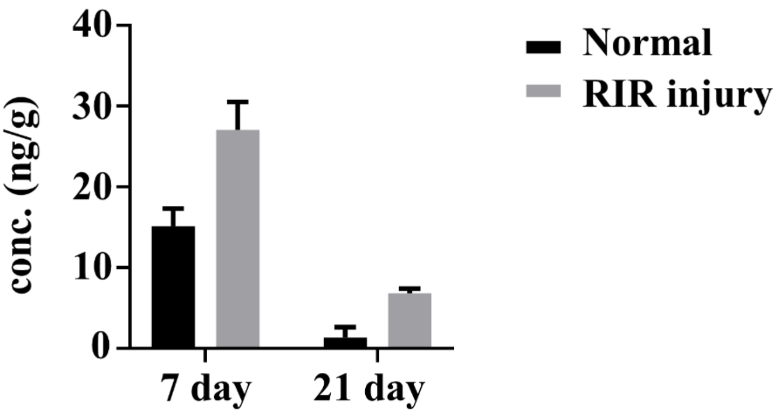

Figure S4. Distribution of rhein in normal and ischemia retinas. Concentrations of rhein in normal and RIR injury retinas after 7 days and 21days following intravitreal injection of Rh-GFFYE.

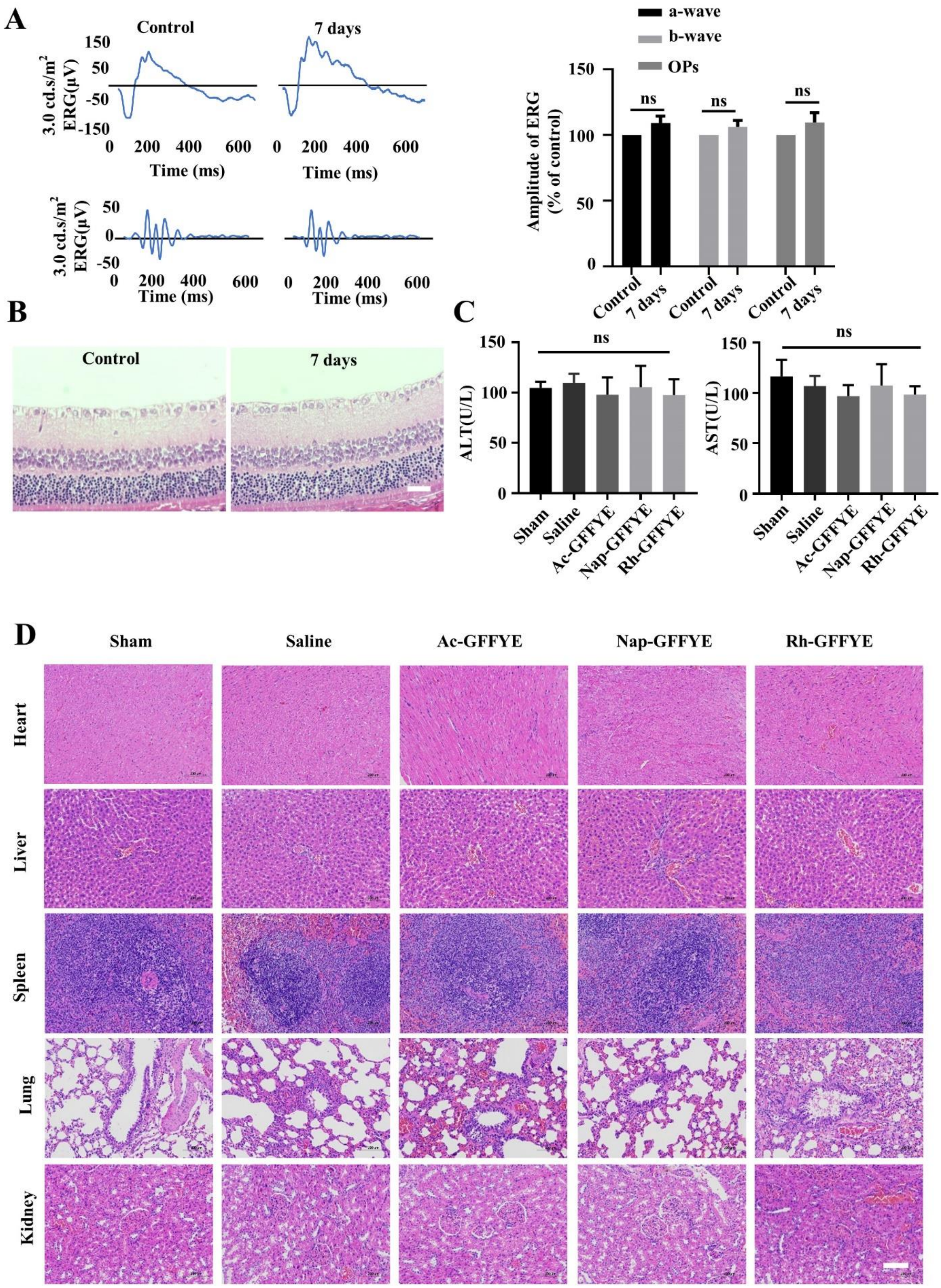

Figure S5. Safety evaluation of Rh-GFFYE. A) Representative ERG traces of normal rats treated with Rh-GFFYE after 7 days (lighting intensity = 3.00 cd·s·m^{-2}), Amplitudes of the scotopic ERG a and b waves and sums of the OPs amplitudes at the light intensity of 3.00 cd·s·m^{-2}. The data were presented as the mean ± SD of n = 6. B) H&E stained retina tissues collected at 7 days after intravitreal injection of Rh-GFFYE. Scale bar = 50 μm. C) Measurement of serum AST and ALT activity in rats after intravitreal injection Rh-GFFYE for 7 days. The data were presented as the mean ± SD of n = 6. D) H&E staining of main organs of heart, liver, spleen, lung, kidney. Scale bar = 100 μm.

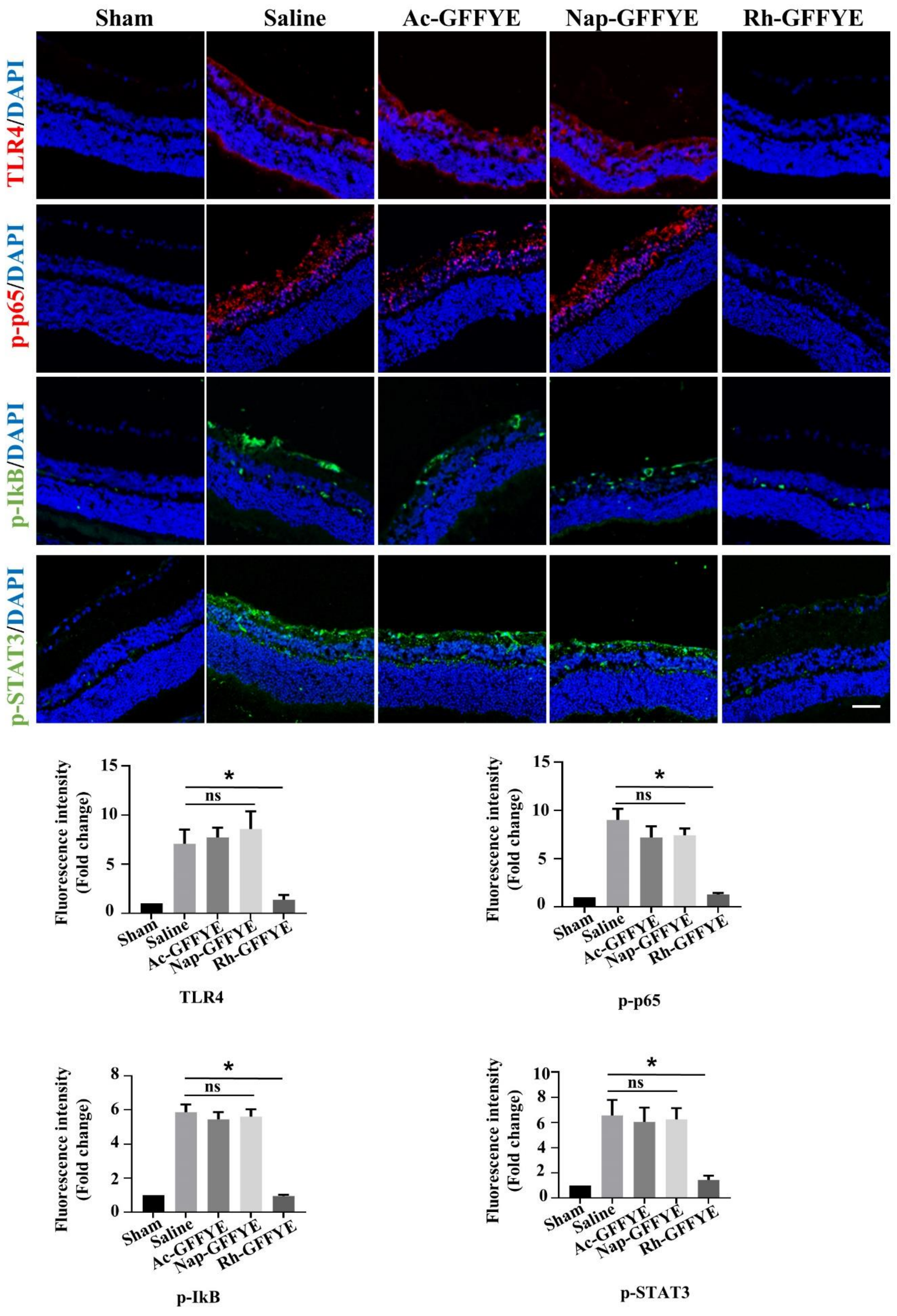

Figure S6. Inhibition of RIR injury-induced upregulation of NF-κB and STAT3 signaling pathways by Rh-GFFYE following intravitreal injection. Representative CLSM images of TLR4, p-NF-κB p65 (p-p65), p-IκB and p-STAT3 in retinal cryosections indicating that Rh-GFFYE-mediated reduction in the level of TLR4, p-

NF-κB p65, p-IκB and p-STAT3 in RIR-injuried retina compared to Saline group. Fluorescence intensities of TLR4, p-NF-κB p65, p-IκB and p-STAT3 quantified by Image J software. Scale bar = 50 μm. The data were presented as the mean ± SD of n = 6. *p < 0.05 compared with the Saline group.

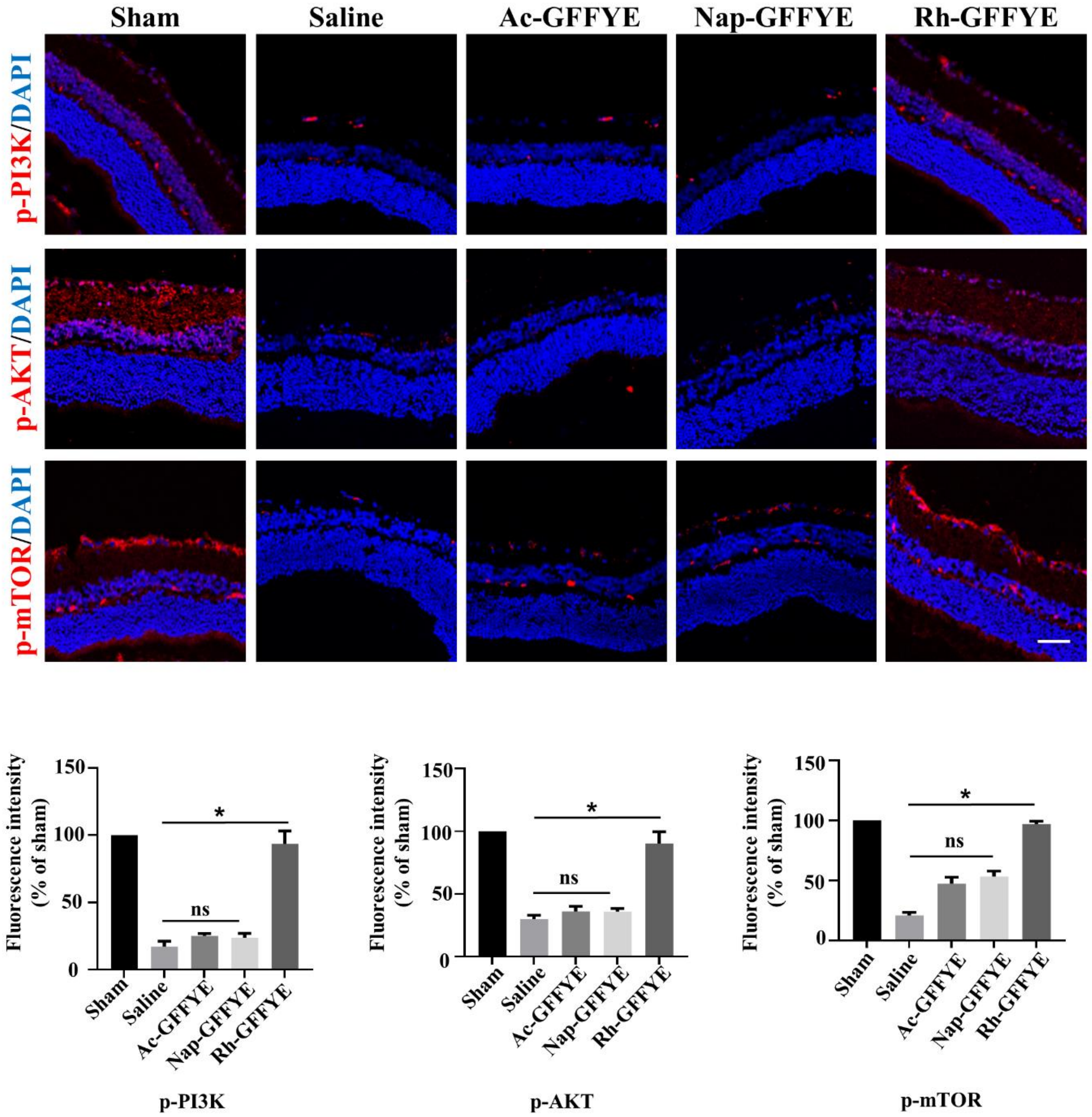

Figure S7. Upregulation of RIR injury-induced inhibition of PI3K/AKT/mTOR signaling pathway by Rh-GFFYE following intravitreal injection. Representative CLSM images of p-PI3K, p-mTOR and p-AKT in retinal cryosections indicating that Rh-GFFYE-mediated enforcement in levels of p-PI3K, p-AKT and p-mTOR in RIR-injuried retina compared to Saline group. Fluorescence intensities of p-PI3K, p-AKT and p-mTOR quantified by Image J software. Scale bar = 50 μm. The data were presented as the mean ± SD of $n = 6$. $^{*}p < 0.05$ compared with the Saline group.
